# Frustration-Induced Many-Body Degeneracy in Spin −1/2 Molecular Quantum Rings

**DOI:** 10.1021/jacs.5c03112

**Published:** 2025-07-11

**Authors:** Donglin Li, Nan Cao, Marvin Metzelaars, Orlando J. Silveira, Joakim Jestilä, Adolfo Fumega, Tomohiko Nishiuchi, Jose Lado, Adam S. Foster, Takashi Kubo, Shigeki Kawai

**Affiliations:** † Center for Basic Research on Materials, National Institute for Materials Science, 1-2-1 Segen, Tsukuba, Ibaraki 305-0047, Japan; ‡ Department of Applied Physics, 174277 Aalto University, Espoo 02150, Finland; § Department of Chemistry, Graduate School of Science, 13013 Osaka University, Toyonaka 560-0043, Japan; ∥ Jülich-Aachen Research Alliance (JARA-FIT) and Institute of Inorganic Chemistry, 9165 RWTH Aachen University, Aachen 52056, Germany; ⊥ Department of Physics, Nanoscience Center, University of Jyväskylä, Jyväskylä 40014, Finland; # Nano Life Science Institute (WPI-NanoLSI), Kanazawa University, Kanazawa 610-101, Japan; ∇ Graduate School of Pure and Applied Sciences, University of Tsukuba, Tsukuba 305-8571, Japan

## Abstract

Frustrated spin systems, where competing interactions prevent conventional magnetic ordering, provide a platform for uncovering emergent quantum phases and exotic many-body phenomena. Particularly, low-dimensional and symmetric geometries without boundary conditions allow us to study unconventional spin states. Here, we present *S* = 1/2 antiferromagnetic Heisenberg cyclic pentamer and hexamer via homocoupling of air-stable phenalenyl derivatives on Au(111). With a combination of scanning tunneling microscopy (STM)/scanning tunneling spectroscopy (STS) at 4.3 K and comprehensive theoretical simulations, we found that while large magnetic exchange interactions exist in both rings, the pentamer features an increased geometric frustration of the system. This frustration induces rotational symmetry in the spin wave function, leading to a 4-fold degenerate ground states of the pentamer. The interplay between molecular geometry and magnetic interactions creates a unique quantum spin environment. Our findings offer a powerful approach for constructing spin-frustrated molecular architectures, allowing precise control over quantum magnetic interactions.

## Graphical abstract

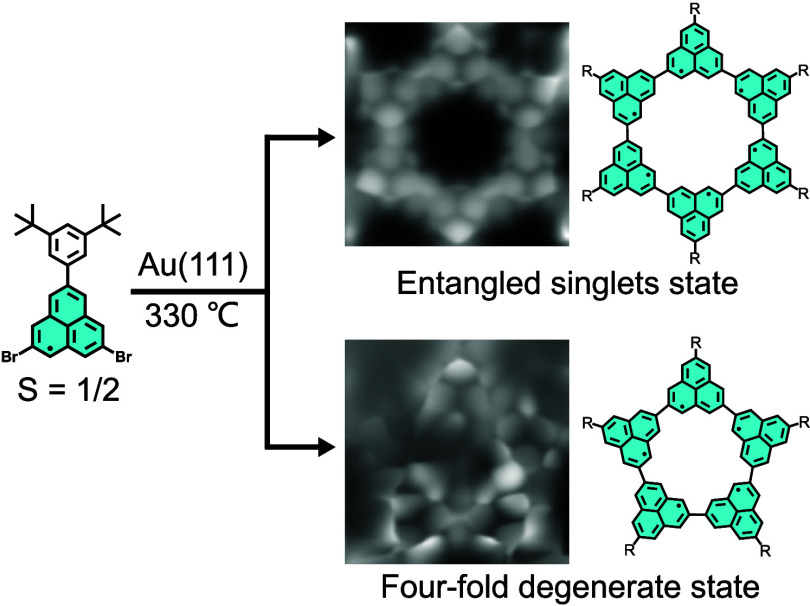

## Introduction

The design and characterization of low-dimensional magnetic systems is of central importance in unraveling the complexities of strongly correlated physics and exploring exotic quantum phenomena, with the enhanced quantum fluctuations facilitating the emergence of universal magnetic properties.^1,2^ Theoretical studies with exact analytical solutions^3^ and numerical renormalization-group methods^4^ have been conducted to understand a multitude of collective phenomena in strongly correlated systems, such as fractionalized spin excitations,^5,6^ quantum spin liquids,^7,8^ spin-Peierls transition,^9–11^ and hidden topological order.^12,13^ Experimental studies were also performed to explore such phenomena in low-dimensional systems of transition metal ions^14^ via neutron scattering,^15^ electron spin resonance,^16–18^ nuclear magnetic resonance,^19^ and thermodynamic property measurements.^20,21^ Despite the significant progress, there are still many uncertainties, such as identifying the ground state of the near-neighbor Heisenberg model and determining the multiple exchange parameters among these models. A persistent challenge in the study of quantum magnetism is the impact of chemical disorder, which promotes an urgent need to explore new synthesis routes.

Scanning tunneling microscopy (STM) offers the possibility to create spin arrays of atoms^22,23^ and molecules on surfaces with atomic precision and consequently investigate spin excitations at the atomic level. Recent advances in on-surface synthesis have enabled the fabrication both chain^24^ and ring^25^ carbon-based π electron magnets with *S* = 1 units. These magnets exhibit long-range spin interactions with different exchange coupling values, which play a crucial role in their unique quantum magnetic states. Unlike magnets with *S* = 1 units, systems with *S* = 1/2 units, characterized by a given exchange coupling, would exhibit even stronger quantum entanglement and longer-ranged quantum correlations. This leads to exotic quantum phenomena, such as spin liquids and spin-Peierls transitions. Therefore, constructing *S* = 1/2 antiferromagnetic Heisenberg model systems with π electron magnets presents a promising avenue for exploring quantum magnetism and investigating both ground states and magnetic excitations. To this end, *S* = 1/2 antiferromagnetic Heisenberg chains were very recently synthesized on surface.^26–31^ They revealed the gapped nature of bulk excitations and the effect of chain parity on their magnetic properties. Naturally, unlike chains magnets, an *S* = 1/2 antiferromagnetic Heisenberg ring with a given exchange coupling is anticipated to exhibit unique spin frustration and nontrivial ground states, attributed to the absence of boundary conditions and the presence of rotational symmetry.

Here, we present on-surface synthesis of quantum rings with *S* = 1/2 [2]­triangulene units under ultrahigh-vacuum (UHV) conditions, with detailed characterization using bond-resolved (BR)-STM and scanning tunneling spectroscopy (STS) at 4.3 K. Experimental characterization reveals that both the hexamer and pentamer exhibit symmetric stepped features around Fermi level in their differential conductance (d*I*/d*V*) spectra. By modeling these systems with Heisenberg Hamiltonians and performing exact diagonalization, we determined that the hexamer exhibits antiferromagnetic coupling between neighboring spins, leading to a global singlet ground state. In contrast, the pentamer ring, with its odd number of *S* = 1/2 units, shows complex magnetic behavior due to geometry frustration, resulting in a 4-fold degenerate ground state influenced by its rotational symmetries.

## Results and Discussion

The quantum ring system composed of *S* = 1/2 units was obtained with sequential solution and on-surface synthesis (Figure 1a,b). Initially, we synthesized the radical 5,8-dibromo-2-(3,5-di-*tert*-butylphenyl)-[2]­triangulene (**1**) in solution; the detailed process is provided in the Methods section. The bulky 3,5-di-*tert*-butylphenyl group was introduced to increase the solubility and sterically protect the reactive site. It is notable that radical **1** features a relatively high stability under ambient conditions with no appreciable decomposition in the solid state and a half lifetime of 42 h in the solution state as determined by ultraviolet–visible (UV–vis) spectroscopy (Figure S1). Furthermore, 3,5-di-*tert*-butylphenyl is expected to facilitate the synthesis of cyclic rings. Radical **1** was then deposited onto a Au(111) substrate kept at 330 °C under UHV conditions. Several ring and linear oligomers were formed via debrominative homocoupling (Figure S2). Here, we focus on the ring-like structures. The corresponding close-up views of the STM topography show the formation of the cyclic hexamer and pentamer, identified by the distinct number of lobes attributed to the bulky 3,5-di-*tert*-butylphenyl groups (Figure 1c,d). To investigate the inner structures, the central cores indicated by green squares were imaged by BR-STM with a CO-functionalized tip.

**Figure 1:**
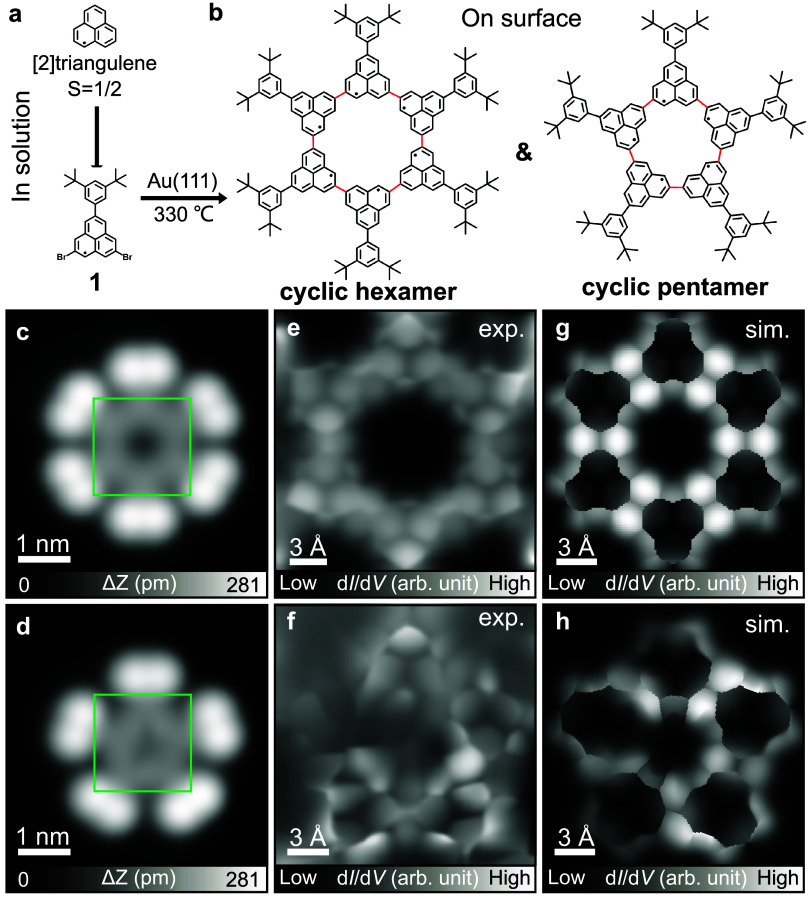
On-surface synthesis of cyclic [2]­triangulene pentamer and hexamer. Chemical structures of (a) [2]­triangulene and its derivative **1** and (b) cyclic hexamer and pentamer. (c, d) Close-up views of STM topographies of hexamer ring and pentamer ring, respectively. (e, f) Corresponding constant-height BR-STM image taken in the areas indicated by green squares in (c, d), respectively. (g, h) Simulated constant-height d*I*/d*V* maps with a relaxed CO tip corresponding to (e, f), respectively. To better resolve the features of the central cyclic hexamer and pentamer, the simulations were performed with [2]­triangulene cyclic hexamer and pentamer models without including the bulky end groups. The cyclic pentamer was simulated based on Structure 8 searched by BOSS (Figure S4). Measurement parameters: Sample bias voltage *V* = 200 mV and tunneling current *I* = 10 pA in (c, d), and *V* = 1 mV in (e, f).

The BR-STM image of the cyclic hexamer reveals a flat structure with a pore comprising six [2]­triangulene units in a closed ring configuration (Figure 1e). In contrast, the cyclic pentamer exhibits an apparent contrast variation within the [2]­triangulene ring (Figure 1f). To investigate the origin of the contrast variation in the triangulene rings, we performed density functional theory (DFT) calculations. Structural optimizations suggest that the hexamer has a planar adsorption geometry on Au(111), while the pentamer adopts a nonplanar geometry upon adsorption (Figure S3). To determine the preferred structural geometry of the pentamer on the surface, we performed an adsorption configuration search based on Bayesian Optimization Structure Search (BOSS).^32^ Our results reveal that the nonplanarity of the pentamer is caused by the geometric frustration between the units (Figure S4), directly causing the observed contrast differences also present in the simulated images in Figure 1g,h. The simulated BR-STM image for hexamer in Figure 1g shows clearly resolved [2]­triangulene units with uniform contrast for all of the units, consistent with the planar geometry imaged experimentally (Figure 1e). While the simulated pentamer in Figure 1h displays a similar contrast variation to the experimentally observed pentamer, with the unit in the upper-left corner appearing relatively darker.

Each [2]­triangulene unit in these ring structures hosts one unpaired π electron, which gives rise to a spin *S* = 1/2 ground state. This is confirmed by STM tip manipulation experiments, where the spin in individual triangulene units can be quenched sequentially via tip-induced dehydrogenation, leaving only one active unit. Tip-induced dehydrogenation of the final hydrogen on the carbon site can be achieved by applying a high bias voltage of approximately 3.3 ± 0.2 V using an STM tip. After dehydrogenation, the carbon forms a bond with a gold atom from the substrate, and the resulting hybridization and charge transfer effectively eliminate the unpaired π-electron.^30^ The presence of a spin-1/2 in this unit is further evidenced by measuring the conductance spectra, with a Kondo resonance appearing at zero bias (Figure S5). This suggests that unpaired spins interact throughout the conjugated triangulene rings and stabilize a collective spin state. In addition, antiferromagnetic coupling between the two nearest neighbors is further confirmed by artificially forming a dimer using tip-induced dehydrogenation (Figure S6).

Both spin-polarized DFT (Figure 2a) and mean-field Hubbard (MFH) simulations (Figure 2b) (see the Methods section for details) reveal that the spin *S* = 1/2 in each triangulene unit is strongly coupled to its neighbors in an antiferromagnetic configuration (Figure S7). Such an interaction pattern is expected to lead to a global spin-singlet ground state (*S*_T_ = 0). Our DFT calculation of the free-standing hexamer ring predicts that the antiferromagnetic *S* = 0 ground state is lower in energy by 81 meV than its *S* = 1 spin states (Figure S7). In addition, we also carried out fully relaxed DFT calculations of the hexamer ring on Au(111), which confirms the energetically preferred antiferromagnetic ground state of the hexamer upon adsorption. The calculated spin density of the structure on the surface shows a very similar character to that of the free-standing structure, as shown in Figure 2a. Similarly, MFH simulations reveal a preferred antiferromagnetic ground state of the hexamer ring and a total energy difference between *S* = 1 and *S* = 0 states of up to 78 meV when *U* = 1.8t (Figure S8). Both density functional theory and tight binding Hubbard calculation treat the spin at the mean-field level. However, including quantum fluctuations in the associated Heisenberg model leads to an entangled singlet ground state, featuring a superposition of pairwise singlets in the hexamer.^33,34^ This entangled ground state has a lower energy than the mean-field symmetry-broken antiferromagnetic state and becomes the ground state of the hexamer. In the hexamer ring, the antiferromagnetic coupling of the nearest-neighbor spins leads to a ground state where the dominating singlets are nearest neighbors.

**Figure 2:**
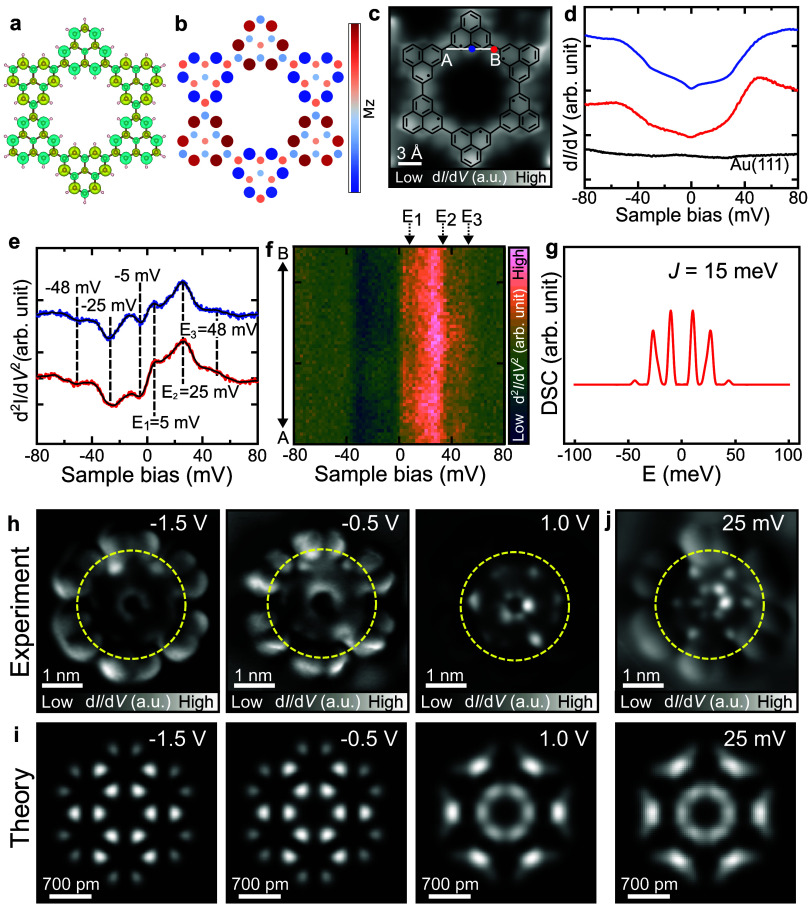
Magnetic excitations and electronic structure of the cyclic [2]­triangulene hexamer. (a) DFT calculated spin density distribution for the [2]­triangulene hexamer ring with a preferred antiferromagnetic singlet ground state. Green and yellow represent the areas with predominant spin-up and spin-down electron densities, respectively. (b) Spin density map of the [2]­triangulene hexamer ring obtained from mean-field Hubbard simulations. *M*z represents the normalized local spin density along the *z*-direction at each site. (c) BR-STM image of the cyclic hexamer with its corresponding chemical structure superimposed. (d) d*I*/d*V* spectra and (e) d^2^ *I*/d*V* ^2^ spectra recorded at the sites indicated by red and blue dots shown in (c), with their corresponding Gaussian-filtered curves (black curves) overlaid to aid in identifying the peak positions. d^2^ *I*/d*V* ^2^ spectra showing inelastic steps at *E*_1_ = 5 mV, *E*_2_ = 25 mV, and *E*_3_= 48 mV. The curves in (d) and (e) are vertically shifted for clarity. (f) Two-dimensional map composed of a series of d^2^ *I*/d*V* ^2^ spectra taken along A-B in (c). (g) Computed dynamical spin correlator with nearest-neighbor interactions *J* = 15 meV and a broadening parameter δ = 5 applied to account for spectral resolution. The resulting spectra reproduce the spin excitations at energy levels comparable to those experimental ones. The influence of second-nearest-neighbor interactions is minor and is discussed in detail in Figure S16. (h) Constant-current d*I*/d*V* maps of the hexamer measured with a CO tip at different bias voltages. (i) DFT simulations of the inner hexamer ring at different energy levels align with experimental data. Here we compare these electronic states with those features distributed on the inner hexamer rings, indicated by the dashed yellow circles in the d*I*/d*V* maps in (h). (j) Spin excitation d*I*/d*V* maps and their corresponding simulations obtained by modulating the DFT calculated d*I*/d*V* map at 0 mV with the DSC at 25 mV. Measurement parameters: (c) *V* = 1 mV, lock-in zero-to-peak modulation voltage, *V*_mod_ = 10 mV. (d–f) Tip–sample gap was adjusted with *V* = 80 mV and *I* = 200 pA before the spectroscopic measurement. *V*_*mod*_ = 2 mV. (h, j) *I* = 100 pA, *V*_*mod*_ = 10 mV.

To experimentally determine the spin configuration of the hexamer, we probed their excitation spectrum using STS. Specifically, we measured the d*I*/d*V* spectra at the edges of the [2]­triangulene units and the bridge sites, as indicated by the blue and red dots in Figure 2c. Both spectra show dip features at zero bias (Figure 2d), indicating the presence of the singlet ground state,^35–37^ consistent with our DFT and MFH simulations. The dip feature was observed in all triangulene units with a similar spectral shape (Figure S9). This indicates that the hexamer is composed of six intact units that hybridized with substrates while retaining the spin (*S* = 1/2). Upon close inspection, these dip features have symmetric steps, which are a characteristic fingerprint of the spin excitation induced by inelastic electron tunneling.^38,39^ The steps can be seen in the d^2^ *I*/d*V* ^2^ spectra as three features at *E*_1_ = ±5 mV, *E*_2_ = ±25 mV, and *E*_3_ = ±48 mV (Figure 2e). To explore the spatial distribution of the spin excitations, a series of d*I*/d*V* spectra were recorded along A to B (Figure 2f). Each of the observed peaks (*E*_1_, *E*_2_, and *E*_3_) is equally spaced relative to the Fermi level and displays nearly uniform intensity across the measured positions. This spectral uniformity suggests that the inelastic signals arise from coherent superpositions of local spin states distributed over the ring. We attribute this behavior to the formation of highly entangled quantum states resulting from the coherent superposition of paired singlet states (Figure S10).

To investigate the three spin excitations, we modeled the spin system with a Heisenberg Hamiltonian *Ĥ* = *J*∑*_i_S⃗*_*i*_ *S⃗*_*i*+1_ (here, *S*_i_ denotes the spin-1/2 operator at site *i* and exchange coupling *J* > 0), describing the nearest-neighbor antiferromagnetic interactions within six *S* = 1/2 spins in a ring. The many-body ground state of the hexamer, obtained by the exact diagonalization methods of the spin Hamiltonian, is a singlet state and consists of the superposition of the classical solutions of antiferromagnetic configuration (i.e., ground state from the DFT/MFH model in Figure 2a,b), and other spin configurations with a global spin *S*_T_ = 0. The excited states are collective spin modes characterized by a total spin number from *S*_T_ = 0 to *S*_T_ = 3, described as a superposition of multiplet states of *S* = 1/2 within the triangulene ring (detailed in Figure S11). By solving a full dynamical spin correlator (DSC), summing over all ground states and all the spin components (see Notes S1–S3 for details), we computed the excitation spectra with the nearest-neighbor interaction *J* = 15 meV. The simulation reveals spin excitations at energy levels of 10, 25, and 48 meV, consistent with the experimental observations (Figure 2g). It is worth noting that the simulated spectra are identical at all units. The consistent spectra observed at all spin units suggest global spin excitations within the cyclic hexamer, as shown by the DSC plots at all units in Figure S12. In addition, we employed a composite spin operator of *S*_*n*_^+^ + λ*S*_*n*+1_^+^ to model the dynamical response in the DSC computation (Figure S13). This is based on the physical consideration that the inelastic electron tunneling is likely to probe not only a single spin center but also its nearest neighboring sites, due to cross-tunneling to modes between adjacent units. This procedure results in spectral features that are comparable with experimental observations, indicating the relevance of the nearest-neighbor contributions to the excitation.

In addition to the observation of the inelastic excitation behavior near zero bias, we explored the spatial distribution of electronic states over a broader energy range of the hexamer. Differential conductance maps were taken at sample biases corresponding to the peaks identified in the wide-range STS curves (Figure S9). These d*I*/d*V* maps show that the electronic states are mainly localized on the inner [2]­triangulene ring (as indicated by the dashed yellow circles in Figure 2h). We performed spin-polarized calculations to examine the frontier molecular orbitals at different energy levels, both for the inner triangulene ring and the hexamer carrying nonplanar bulky groups. The results suggest that the bulky groups do not significantly contribute to the electronic states at the observed energies (Figure S14). Therefore, we performed simulations for the d*I*/d*V* maps based on the inner triangulene ring. The simulations exhibit consistent features acquired at biases between −2.0 and 0.6 V, where the molecular orbitals are primarily localized at the edges of the triangulene units (Figures 2i and S15). At higher energies, specifically above 1.0 V, electronic states become more pronounced toward the center of the hexamer. The overall d*I*/d*V* maps exhibit an invariant spatial distribution of the electronic states in an energy range of −2.0 to 2.0 V. DFT simulations for the triangulene ring reproduce this spatial distribution of molecular orbitals, corroborating the experimental data. Apart from these electronic states, the spin excitation d*I*/d*V* map collected at 25 mV (Figure 2j) also shows consistent features over all of the units, indicating the global spin excitations.

An intuitive picture of the spin state in the cyclic [2]­triangulene pentamer, composed of five *S* = 1/2 spins, leads to a ground state with *S*_T_ = 1/2, as the odd-numbered spins cannot fully pair into singlets at once. This configuration can be reflected in the differential conductance spectra, where Kondo resonances typically appear due to the screening effect of the substrate conduction electrons. However, our d*I*/d*V* spectra taken at the edges of each unit (indicated by colored dots in Figures 3a and S17) show dip features around zero bias, resembling those observed in the cyclic hexamer (Figure 3b). The absence of Kondo resonances in the pentamer suggests that the system does not exhibit a simple localized *S* = 1/2 magnetic moment interacting with conduction electrons. Instead, it points to a more complex, many-body quantum state. It is worth noting that the odd number closed chain has geometric frustration due to its antiferromagnetic coupling, a feature that leads to a 4-fold degenerate ground state.

**Figure 3:**
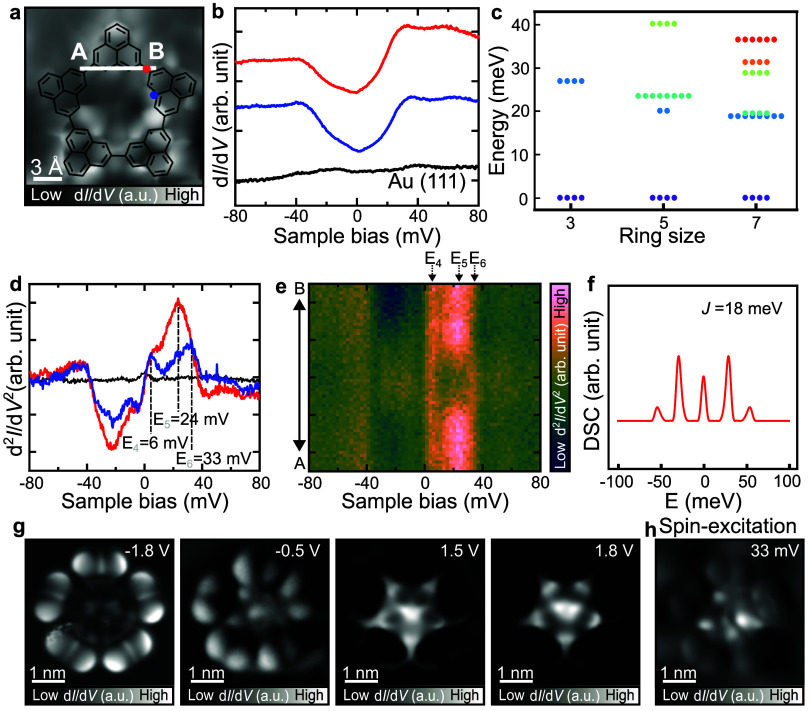
Spin coupling in cyclic [2]­triangulene pentamer and its magnetic and electronic properties. (a) BR-STM image of the pentamer with its corresponding chemical structure superimposed. (b) d*I*/d*V* spectra taken at the pentamer sites indicated by red and blue dots in (a) as well as on the bare Au(111) surface for a reference. The curves in (b) are vertically shifted for clarity. (c) Calculated ground states and excited states below 40 meV for spin rings containing 3, 5, and 7 spin-1/2 units, obtained from a Heisenberg model with *J* = 18 meV with closed boundary conditions. This plot illustrates the general trend of ground state degeneracy in odd-numbered spin rings. Each color represents a distinct energy level, and the number of dots at each energy indicates the degeneracy of that state. For example, the four purple dots at the lowest energy level represent the 4-fold degenerate ground state, the blue dots correspond to the first excited states. (d) d^2^ *I*/d*V* ^2^ spectra taken at the pentamer sites indicated by red and blue dots in (a) as well as on the bare Au(111) surface for a reference. The inelastic steps were at *E*_4_ = 6 mV, *E*_5_ = 24 mV, and *E*_6_= 33 mV. (e) Line profile d^2^ *I*/d*V* ^2^ spectra measured along the [2]­triangular unit indicated by the white lines A-B in (a). (f) Computed full dynamical spin correlator reproduces the spin excitations at energy levels comparable to the experimental ones. A broadened parameter δ = 5 was used to plot the DSC spectra. (g) Constant-current d*I*/d*V* maps of the pentamer using a CO tip at different biases. (h) Spin excitation d*I*/d*V* maps at 33 mV. Measurement parameters: (a) *V* = 1 mV and *V*_mod_ = 10 mV. (b, d, e) The tip–sample gap was adjusted with *V* = 80 mV and *I* = 200 pA each before taking the spectroscopic curve at the corresponding measurement sites in (a). *V*_mod_ = 2 mV. (g, h) *I* = 100 pA and *V*_mod_ = 10 mV.

To understand the many-body ground spin states in the pentamer, we computed the Heisenberg Hamiltonian for a system of five spin-1/2 units arranged in a closed ring by exact diagonalization (Figure 3c). The calculations reveal that the cyclic pentamer holds a 4-fold degenerate ground state (indicated by four purple dots in Figure 3c), arising from superpositions of spin configurations that contribute to a total spin *S*_T_ = 1/2. A complete excitation energy spectrum is provided in Figure S11. The dip features observed over each unit indicate that all units are intact and retain spin despite differences in their degree of hybridization with the substrate. The cyclic structure of the pentamer exhibits a 5-fold rotational symmetry (*C*_5_), which corresponds to the translation symmetry of the spin Hamiltonian under periodic boundary conditions. This implies that the translated Hamiltonian remains invariant under a cyclic transformation of the spin positions. Mathematically, this invariance indicates that the spin Hamiltonian commutes with the translation operator: *THT* ^–1^ = *H*. In other words, Hamiltonian *H* and translation operator *T* share the same eigenstates. Consequently, the eigenstates of *H* can be labeled by eigenvalues of *T*. The translation operator *T* acts on the spin wave function by swapping neighboring spins, described as T|Ψ⟩ = e^*i*ϕ^|Ψ⟩, where the geometric phase ϕ is determined by discrete rotations of the system. For a pentamer (*L* = 5), the phase is given by $\phi =\frac{2\pi C}{L}=\pm \frac{2\pi }{5}$ , here *C* = ±1 is an internal quantum number that can be understood as a pseudospin degeneracy, reflecting two distinct states with opposite “twists” in the spin wave functions. Thus, the interplay between spin Hamiltonian and translation symmetry results in a ground state manifold featuring four states with *S*_T_ = 1/2, two with *S*_*z*_ = 1/2 (*C* = ±1), and two with *S*_*z*_ = −1/2 (*C* = ±1) (detailed in Supporting Note S2). This multidegeneracy reflects an interaction between the spin frustration (due to the odd-numbered spins) and an additional internal quantum degree of freedom due to the rotational symmetry of the system (see also the discussion in Figure 4). Further analysis of the experimental spin excitations, specifically the d*I*/d*V* spectra, reveals distinct conductance steps at higher energy levels. These steps became particularly clear in the d^2^ *I*/d*V* ^2^ spectra at *E*_4_ = ±6 and *E*_6_ = ±33 mV (Figure 3d). We found that the magnetic exchange interaction at the bridge site (*E*_5_ = ±24 mV, the red dot in Figure 3a) is weaker than that at the triangulene unit (*E*_6_ = ±33 mV, the blue dot in Figure 3a). This difference indicates a stronger spin coupling at the triangulene edges, likely influenced by the nonplanar structural geometry of the pentamer (discussed in Figure 1), where the local environment leads to nonuniform spin interactions. In addition, the d^2^ *I*/d*V* ^2^ spectral map, composed of a series of d^2^ *I*/d*V* ^2^ curves taken along A-B in Figure 3a, shows apparent intensity changes over the triangulene unit (Figure 3e). Such spatial variation can be attributed to the structural distortion of the pentamer, which enhances the frustration of magnetic interactions. As before, by computing the full dynamic spin correlator with a nearest-neighbor exchange coupling of *J* = 18 meV, we were able to reproduce the dominant spin excitations at energies observed experimentally (Figure 3f). In addition to these excitations, we observe a weak zero-bias signal associated with spin-flip scattering within the *S*_T_ = 1/2 ground state manifold of the pentamer ring. This indicates the existence of low-energy spin fluctuations, which in principle could support Kondo screening. However, no corresponding Kondo resonance is detected in the experimental d*I*/d*V* spectra. One possible reason is that the effective Kondo temperature of the pentamer may be very low, as suggested by the weak intensity of the zero-bias peak in the DSC spectra, causing the resonance to fall below the experimental detection limit.^40^ Alternatively, if the effective exchange coupling between the spin ring and the substrate conduction electrons is ferromagnetic, the Kondo effect can be suppressed entirely, resulting in the absence of a zero-bias resonance.^41,42^ The absence of the Kondo resonance in the d*I*/d*V* spectra does not contradict the weak zero-bias signal in the DSC spectra, instead, it reflects the fact that the observation of Kondo resonance requires not only low-energy spin fluctuations but also sufficiently strong antiferromagnetic coupling to the substrate.^43^ In addition, the possibility of a pentamer composed of four intact units and one quenched unit is also considered in the Supporting Information (Figure S18).

**Figure 4:**
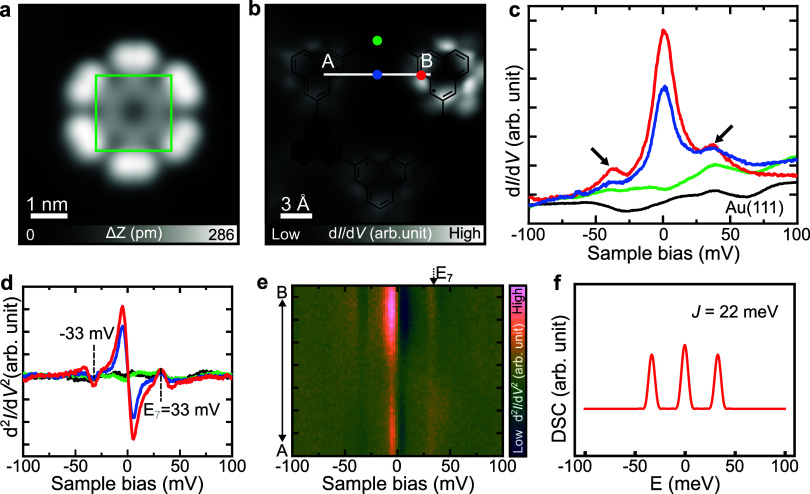
Characterization of the magnetic properties of half-quenched hexamer. (a) STM topography and (b) BR-STM image of the half-quenched hexamer with its corresponding chemical structure superimposed. White units in the chemical structure indicate that they are quenched. (c, d) d*I*/d*V* spectra and the corresponding d^2^ *I*/d*V* ^2^ spectra measured at the unquenched and quenched sites as well as at the site between two unquenched units by red and green as well as blue dots in (b), respectively. The tip–sample gap was adjusted with *V* = 100 mV and *I* = 200 pA before each spectroscopic measurement at the corresponding sites shown in (b). The curves in (c) are vertically shifted for clarity. (e) d^2^ *I*/d*V* ^2^ spectral line measured between two nonquenched units indicated by the white line A-B. (f) The computed dynamic spin correlator reveals the Kondo feature and spin excitations at energy levels aligned with the experiment. A broadening parameter δ = 8 was used. Measurement parameters: (a) *V* = 200 mV, *I* = 10 pA. (b) *V* = 1 mV, *V*_*mod*_ = 10 mV. (c–e) The tip–sample gap was adjusted with *V* = 100 mV and *I* = 200 pA before the spectroscopic measurement. *V*_*mod*_ = 2 mV.

To investigate the spatial distribution of the spin state and electronic properties of the pentamer, constant-current d*I*/d*V* maps were recorded at different energies (Figures 3g,h and S19). Analogous to the hexamer, both occupied and unoccupied maps exhibit a dominant intensity in the triangulene ring regions. In addition, in the unoccupied states, electronic states are also observed at the edges of the ring. In contrast to the hexamer, these variant electronic states in the pentamer could result from slight distortion in the structural geometry.

Following the intriguing analysis of the pentamer, we extend our investigation to another odd-numbered system, a trimer including three spin-1/2 units in a ring arrangement. Resembling the cyclic pentamer, the trimer may exhibit similar quantum behavior and ground state degeneracies driven by its intrinsic rotational symmetry and spin interactions. In the experiment, we found a hexamer ring containing three intact units identical to those in the hexamer shown in Figure 2, while the other three units appear to be quenched, presumably due to strong hybridization with the substrate. This spin-quenching happened every two units, effectively isolating three *S* = 1/2 spins in a cyclic geometry. While the STM topography of the spin-quenched cyclic hexamer is almost identical to those without spin-quenching (Figure 4a), the BR-STM images reveal stark differences. The units carrying spins show brightness, while those that underwent spin-quenching are featureless (Figure 4b). This phenomenon is confirmed by their featureless d*I*/d*V* spectra, indicated by a green dot in Figures 4b and S20.

STS measurements over the bright units reveal spin responses to tunneling electrons. Specifically, the d*I*/d*V* spectra acquired from one of these bright units (indicated by a red dot in Figure 4b) show a prominent zero-bias peak and two symmetrical side-steps (black arrows in Figure 4c and dashed lines in Figure 4d). Similar characteristics were observed in the spectra taken from the other two bright units (Figure S19). To further investigate the spin configuration of the half-quenched hexamer, a series of d^2^ *I*/d*V* ^2^ spectra were taken along the white line shown in Figure 4b crossing two [2]­triangulene units (Figure 4e). The sidestep (indicated by the black dotted arrow) is seen on the whole recorded site, implying that the spin-exchange interaction between them is sufficiently strong. These features resemble those observed in a *S* = 3/2 system (Co on Cu_2_N/Cu­(100)),^44^ leading to an intuitive conclusion that the system hosts a ferromagnetically coupled ground state with *S*_T_ = 3/2. However, our calculations based on the spin Hamiltonian of the trimer indicate a 4-fold degenerate ground state, which consists of superpositions of the spin configurations with a total spin of *S*_T_ = 1/2 (indicated by the four purple dots in Figure 3c). Analogous to our previous analysis for the pentamer, three *S* = 1/2 spins in a trimer system introduce a 3-fold rotational symmetry to the spin Hamiltonian, resulting in an internal quantum degeneracy. The degeneracy gives rise to multiple quantum states arising from different combinations of *S*_*z*_ projections and the internal quantum number *C*. In addition, computing the full dynamic spin correlator of the trimer reproduces spin excitations that are comparable to the experimental data (Figures 4f and S12). Similar to the hexamer, the dynamic spin correlator can also be treated with composite spin operator *S*_*n*_^+^ + λ*S*_*n*+1_^+^ to align with the experimental spectra (Figure S13).

## Conclusions

In summary, we realized *S* = 1/2 antiferromagnetic Heisenberg rings using [2]­triangulene with an on-surface synthesis. Combining STS measurements and theoretical calculations, the cyclic [2]­triangulene pentamer and hexamer feature radically different ground states. For the hexamer, d*I*/d*V* spectra show that it is not a Néel state but a many-body analogue spin-singlet ground state, where the spins are constantly changing their singlet partners. For the pentamer, we observed dip features at zero bias similar to that of the hexamer, including magnetic exchange coupling strength, and spin excitation maps. Our calculations based on the spin Hamiltonian reveal a highly degenerated ground state for the odd-numbered rings. Extending this analysis suggests that the quantum phenomenon observed in the pentamer and trimer spin systems could apply more broadly to other cyclic systems with odd-numbered spin-1/2. For instance, theoretical predictions indicate that a ring with seven *S* = 1/2 spins would exhibit a similar 4-fold degeneracy of the ground state (Figure 3c). Conversely, the *S* = 1/2 spins in even-numbered systems are fully paired into singlets, leading to a highly symmetric, global singlet state (*S*_T_ = 0) without degeneracy. By investigating systems with varying numbers of spin-1/2 units with closed boundary conditions, we may uncover universal features in their spin Hamiltonians. These results ultimately enrich our understanding of quantum magnetism and the role of symmetry in determining ground state properties, and pave the way to explore strongly correlated phases in purely organic systems, such as two-dimensional spin arrays and spintronic devices.

## Methods

### STM Experiments

The experiments were conducted in a homemade low-temperature STM operated under ultrahigh vacuum (*P* < 5 × 10^–10^ mbar) at a temperature of 4.3 K. Chemically etched tungsten STM tip was used. The BR-STM images were acquired by recording the d*I*/d*V* signals in constant-height mode (*V* = 1 mV; *V*_mod_ = 10 mV) using a CO-functionalized tip.^45,46^ The obtained STM images were processed by using Gwyddion software. A clean Au(111) surface (MaTeck GmbH) was prepared through several cycles of Ar+ sputtering for 10 min, followed by annealing at 700 K for 15 min. The sample temperature was monitored by using a thermocouple and a pyrometer. The deposition of precursor molecules onto the clean Au(111) surface was achieved using a Knudsen cell (Kentax GmbH), with the surface held at various temperatures during the deposition process. All the d*I*/d*V* and d^2^ *I*/d*V* ^2^ spectra were taken using a metal tip with a lock-in modulation voltage of 2 mV. The tip–sample gap was adjusted each before taking the spectroscopic curve using bias and current settings, with details provided in the corresponding figure captions. All details of the d*I*/d*V* maps are provided in the corresponding figure captions.

### Instrumentation

Atmospheric pressure chemical ionization (APCI) high-resolution mass spectrometry (HRMS) was performed on a Bruker micrOTOF II spectrometer. Nuclear magnetic resonance (NMR) experiments were performed on a JEOL JNM-ECS400 NMR spectrometer (400 MHz for ^1^H- and 101 MHz for ^13^C NMR). The spectra were recorded in chloroform-d1 and referenced to the residual solvent signals. UV–vis measurements were performed on a Shimadzu UV-2600 spectrophotometer using 10 mm cuvettes with screw caps (QS, Hellman Analytics). The radical solution was prepared in toluene under air and sealed with a screw cap to prevent evaporation of the solvent.

### In-Solution Synthesis of Precursors Radical **1**

The radical 5,8-dibromo-2-(3,5-di-*tert*-butylphenyl)-[2]­triangulene **1** that acts as a precursor for the on-surface cyclization reactions has been synthesized via in-solution synthetic procedures in five steps as summarized in Supporting Scheme S1. The starting material 2-(3,5-di-*tert*-butylphenyl)-2,3-dihydro-1*H*-phenalen-1-one (**2**) has been prepared as previously reported by Hirao et al.^47^ and was directly functionalized at the sterically less demanding carbon atoms at the 5- and 8-positions of the [2]­triangulene core via iridium-catalyzed borylation.^48^ Subsequent bromination using CuBr_2_ ^49^ produced dibrominated intermediate **4a** and 1–5% of oxidized side product **4b** that was readily separated after reducing **4a** to alcohol **5**. The target radical **1** was received by the dehydration of **5** and subsequent reaction with *p*-chloranil.

The identity and purity of all compounds have been verified via ^1^H and ^13^C NMR (Figures S20–24) and APCI-HRMS (Figures S25–29).

### Theoretical Calculations

We performed spin-polarized DFT calculations using the FHI-aims code^50^ employing the B3LYP exchange-correlation functional^51^ for all free-standing molecules. We also carried out fully relaxed DFT simulations of the [2]­triangulene hexamer ring on Au(111) using the PBE functional.^52^ Both calculations use a standard light basis set. The hexamer on Au(111) substrate was modeled including three Au atomic layers with the bottom two layers fully constrained. All of the structural relaxations were carried out until the total energy and remaining atomic forces were less than 10^–6^ eV and 10^–2^ eV/Å, respectively. We used the γ point to sample Brillouin zone for structural optimization and 8 times denser *k*-grid to calculate density of states for the hexamer. The orbital densities of frontier molecular orbitals were obtained from the DFT calculations of free-standing molecules. Bayesian Optimization Structure Search was employed with the MACE-MP-0 foundational model machine learning potential^53^ to determine possible adsorption configurations of the pentamer. More details on this procedure can be found in the Supporting Information.

Constant-height d*I*/d*V* maps were simulated using PP-STM and PPAFM code with flexible CO tips.^54,55^ The PPAFM code was used first to model the positions of the CO tips. The lateral stiffness for the CO tip was set to 0.25 N/m, and an oscillation amplitude of 1.0 Å was used. Subsequently, the d*I*/d*V* maps were generated by PPSTM with various tip models with different orbital compositions, pure *s*-wave, and mixed *s* and *pxy* waves for both hexamer and pentamer rings. A detailed comparison of the simulations using different tips is provided in Figures S15 and S18. The mean-field Hubbard calculations were performed using the PYQULA library,^56^ and the dynamical spin correlators were computed with the DMRGPY library.^57^

## Supplementary Material



## Notes

Materials and methods, solution-based synthetic procedures, NMR spectra and APCI-HRMS measurements of precursor, DFT calculation details, and additional STM data (PDF)
