# Supplementary material for: Frustration-Induced Many-Body Degeneracy in Spin −1/2 Molecular Quantum Rings
Source: J Am Chem Soc. 2025 Jul 11;147(30):26208–17. doi: 10.1021/jacs.5c03112 (PMC12314894; doi:10.1021/jacs.5c03112)
Supplement: Supplementary file 1 [file ja5c03112_si_001.pdf]

Supplementary Materials for

Frustration-Induced Many-Body Degeneracy in Spin -1/2 Molecular Quantum Rings

Donglin Li[a‡], Nan Cao[b‡], Marvin Metzelaars[c,d‡], Orlando J. Silveira[b,e], Joakim Jestilä[b], Adolfo Fumega[b], Tomohiko Nishiuchi[c], Jose Lado[b], Adam S. Foster[b,f*], Takashi Kubo[c*], Shigeki Kawai[a,g*]

[a]Center for Basic Research on Materials, National Institute for Materials Science, 1-2-1 Segen, Tsukuba, Ibaraki 305-0047, Japan.

[b]Department of Applied Physics, Aalto University, Espoo 02150, Finland.

[c]Department of Chemistry, Graduate School of Science, Osaka University, Toyonaka 560-0043, Japan.

[d]Jülich-Aachen Research Alliance (JARA-FIT) and Institute of Inorganic Chemistry, RWTH Aachen University, Aachen 52056, Germany

[e]Department of Physics, Nanoscience Center, University of Jyväskylä, Jyväskylä 40014, Finland.

[f]Nano Life Science Institute (WPI-NanoLSI), Kanazawa University, Kanazawa 610-101, Japan.

[g]Graduate School of Pure and Applied Sciences, University of Tsukuba, Tsukuba 305-8571, Japan.

‡D.L., N.C. and M.M. contributed equally to this work.

*Corresponding author. Email: KAWAI.Shigeki@nims.go.jp; adam.foster@aalto.fi; kubo@chem.sci.osaka-u.ac.jp

This PDF file includes:

Fig. S1. UV-Vis absorbance spectra of **1**.
Fig. S2. Large-scale STM topography.
Fig. S3. DFT optimized models of [2]triangulene cyclic hexamer and pentamer on Au(111).
Fig. S4. BOSS structure search
Fig. S5. Evidence of the spin-1/2 in each triangulene unit
Fig. S6. Nearest-neighbor dimer and next-nearest-neighbor dimer
Fig. S7. Calculated spin density distributions and energy difference of hexamer
Fig. S8. Mean-field Hubbard calculations of [2]triangulene hexamer ring
Fig. S9. d*I*/d*V* spectra of [6]TSR
Fig. S10. Schematic illustration of the formation of the RVB
Fig. S11. Energy diagrams of spin excitation states
Fig. S12. Computed full dynamic spin correlators
Fig. S13. Computed dynamic spin correlators
Fig. S14. Spin-polarized calculations of molecular orbital densities of hexamer
Fig. S15. Experimental and simulated d*I*/d*V* maps of the cyclic hexamer
Fig. S16. Computed full dynamic spin correlators including both J_1 and J_2
Fig. S17. d*I*/d*V* spectra of [5]TSR
Fig. S18. Full dynamic spin correlator of an asymmetric [4[TSR
Fig. S19. Experimental and simulated d*I*/d*V* maps of the cyclic pentamer
Fig. S20. Magnetic and electronic properties of half-quenched [5]TSR
Note S1. Heisenberg Hamiltonian.
Note S2. Origin of the ground state degeneracy.
Note S3. Dynamical spin correlator.
Synthesis of **1**
Fig. S21. 1H- and 13C-NMR spectra of **3** measured in chloroform-d1.

Fig. S22. 1H- and 13C-NMR spectra of **4a** measured in chloroform-d1.
Fig. S23. 1H- and 13C-NMR spectra of **4b** measured in chloroform-d1.
Fig. S24. 1H- and 13C-NMR spectra of **5** measured in chloroform-d1.
Fig. S25. 1H- and 13C-NMR spectra of **1** measured in chloroform-d1.
Fig. S26. Results of APCI-HRMS measurements of **3**
Fig. S27. Results of APCI-HRMS measurements of **4a**
Fig. S28. Results of APCI-HRMS measurements of **4b**
Fig. S29. Results of APCI-HRMS measurements of **5**
Fig. S30. Results of APCI-HRMS measurements of **1**

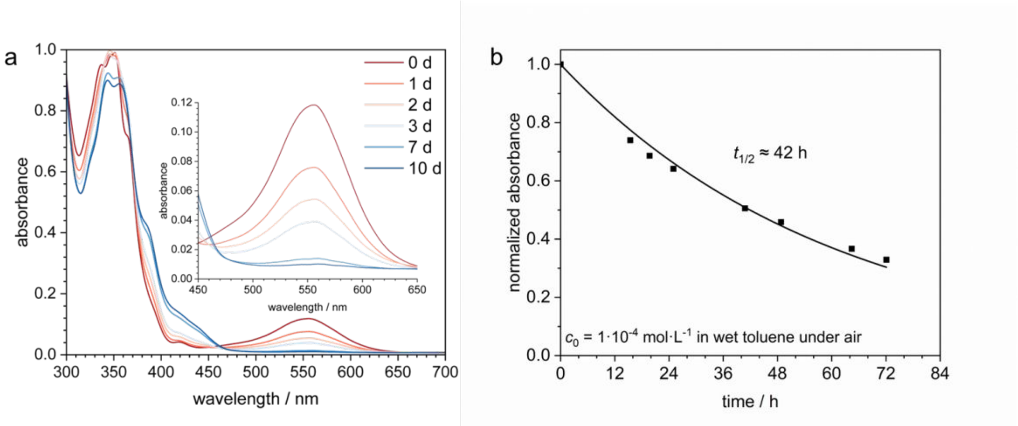

Figure S1. Stability measurement for radical **1** in wet toluene under ambient conditions. (a) UV-Vis absorbance spectra of **1** showing comparably slow decomposition, indicated by the decrease of the peak at 556 nm (inset), which is characteristic for phenalenyl derivatives.[1] (b) Decrease of the peak intensity at 556 nm fitted with an exponential decay function corresponding to a half-life time of 42 h.

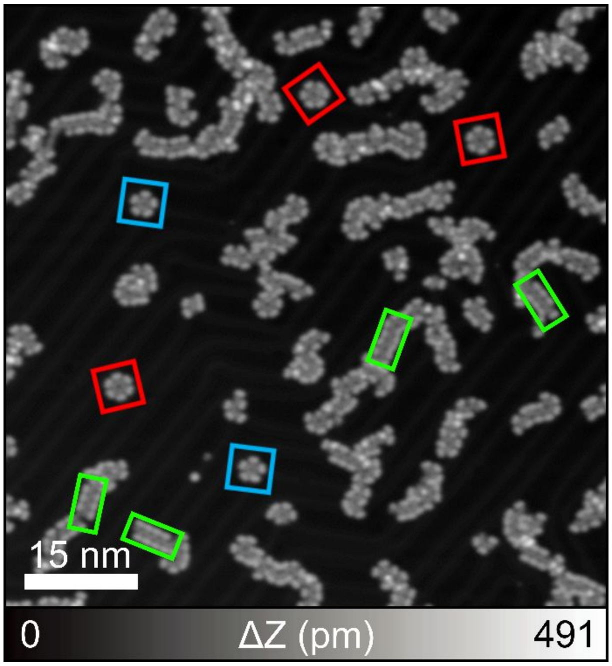

Figure S2. Large-scale STM topography taken after deposition of **1** on Au(111) held at 330 °C. The oligomers, hexamer rings, and pentamer rings are highlighted by green, red, and blue squares, respectively. Measurement parameters: Sample bias voltage V = 200 mV and tunneling current I = 10 pA.

Note that we also tried to deposit precursor **1** on Au(111) kept at room temperature followed by annealing to promote the surface-catalyzed Ullmann-like polymerization, but the yield of ring-like structures is quite low. Deposition of precursor **1** on a hot substrate obviously increases the yield, although 330 °C is higher than the typical temperature for Ullmann coupling.

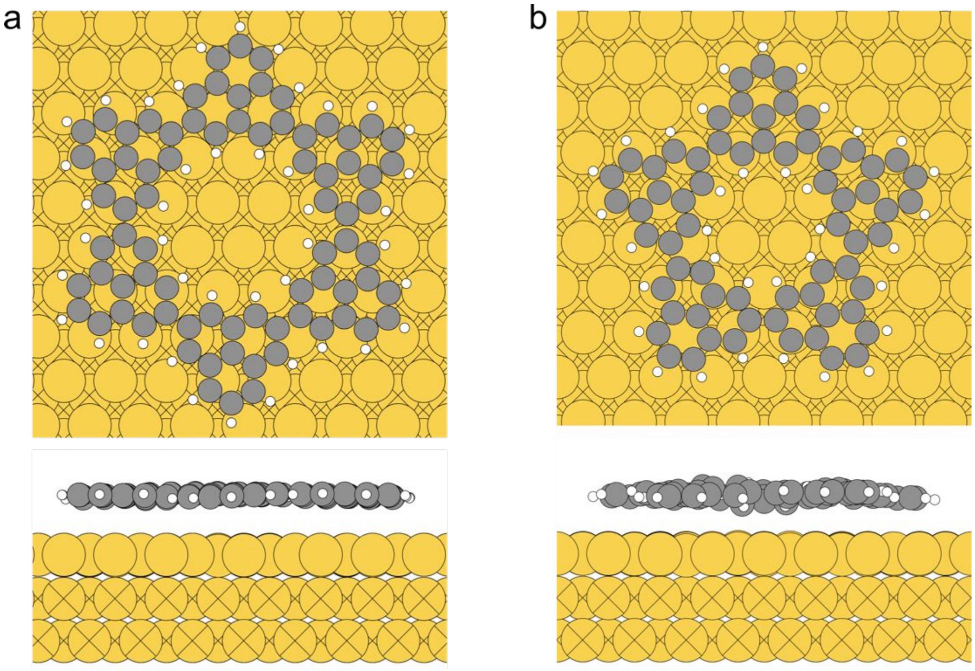

Figure S3. DFT optimized models of [2]triangulene cyclic hexamer and pentamer on Au(111). Structural optimizations show that the hexamer (**a**) has a relatively flat geometry when adsorbed on Au(111), while the pentamer (**b**) adopts a slightly distorted adsorption geometry.

To further elucidate possible pentamer adsorption structures, including the bulky 3,5-ditertbutylphenyl side groups, we employed the Bayesian Optimization Structure Search (*31*). Due to the size of the system, we leveraged the foundational model of the machine learning interatomic potential MACE (MACE-MP), pretrained on Materials Project data (*48*). While BOSS provided preliminary adsorption structures differing mainly in translational and rotational coordinates, a select few of these were refined manually to include variations in the molecular carbon backbone more accurately. The results of this search and the subsequent refinement reveal several possible configurations, showing different puckering orientations of the individual monomers (fig. S4). Due to its sensitivity to height variations, we used simulated AFM images to illustrate these minute structural changes in the pentamer. We note how the strain on the molecule brings certain parts in line at approximately similar heights, resulting in the appearance of triangular shapes in the images (structures 2-4 and 8), similar to what is observed in the experimental BR-STM images of the pentamer. The aforementioned structures were subsequently relaxed with the more accurate DFT method (PBE+vdW^{surf}) as validation.

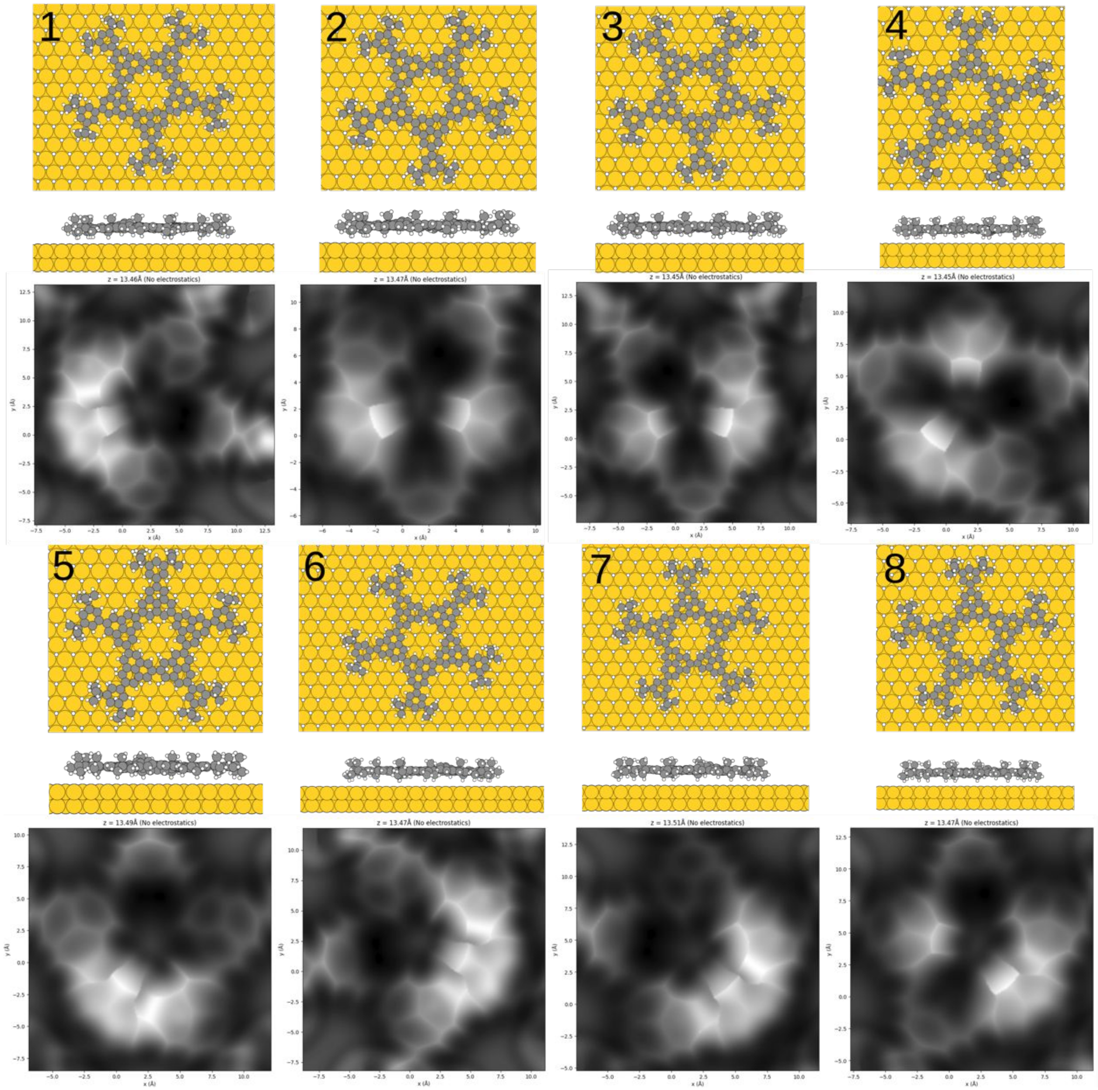

Figure S4. Pentamer adsorption structures (1-8) on Au(111) from Bayesian Optimization Structure Search (BOSS) using the foundational model of the machine learning interatomic potential MACE (MACE-MP). Geometries 1 and 5-8 were obtained from the structure search, while 2-4 were obtained by lowering or lifting given monomers relative to the other units by hand, and subsequently relaxing the full geometry again.

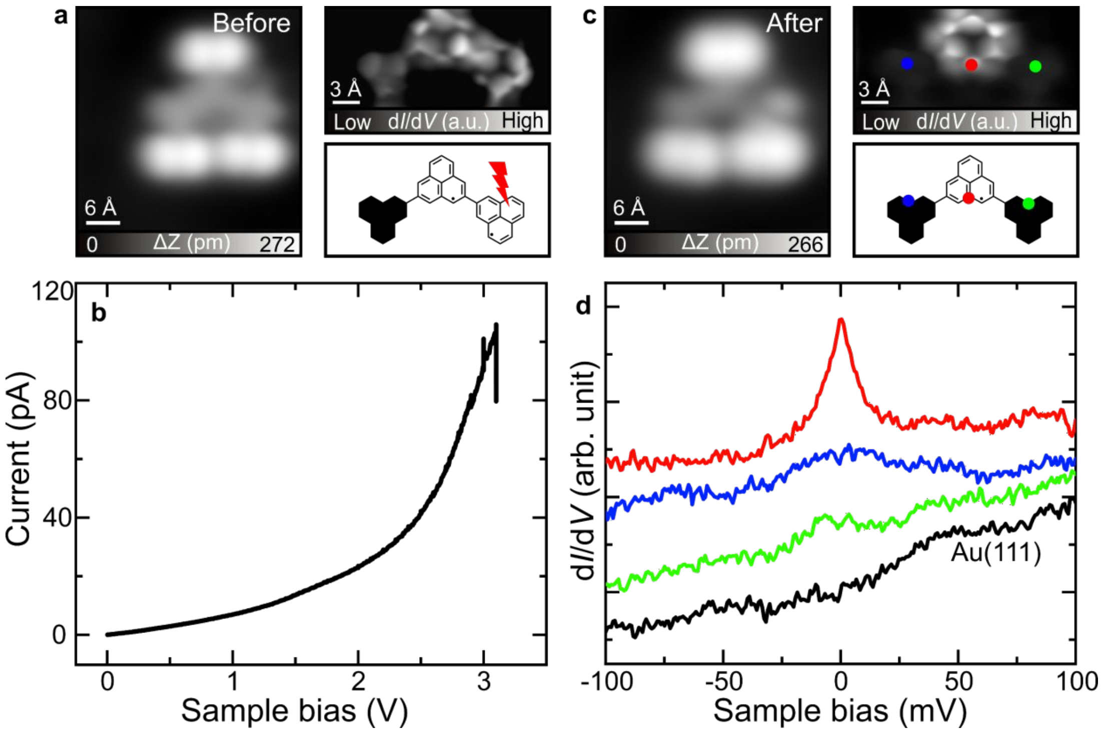

Figure S5. Evidence of spin-1/2 in an individual triangulene unit. (**a**) STM image, BR-STM image, and corresponding chemical structure of a trimer chain before dehydrogenation. The red lightning indicates the dehydrogenation site. (**b**) The I-V curve during the dehydrogenation process. The sudden change of the current indicates that the dehydrogenation happened. (**c**) STM image, BR-STM image, and corresponding chemical structure of a trimer chain after dehydrogenation. (**d**) dI/dV spectra recorded at the positions marked by red, green, and blue dots in (b), along with a reference spectrum from Au(111). Lock-in amplitude: 2 mV. Sample bias: 100 mV. Setpoint: 200 pA.

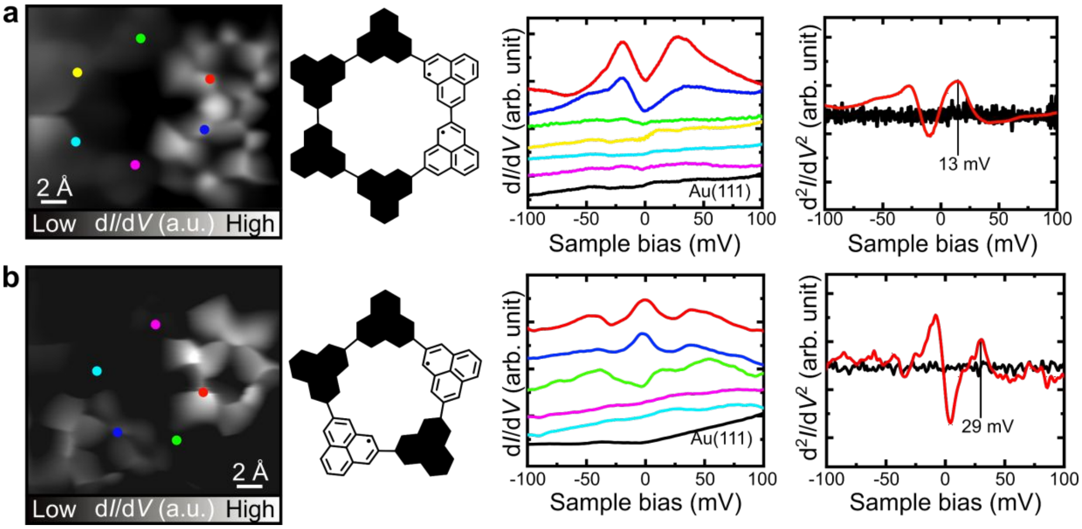

Figure S6. Two dimers, one with nearest-neighbor (dimer 1) and the other with next-nearest-neighbor (dimer 2) configurations, were fabricated through tip-induced dehydrogenation. (a) BR-STM image of dimer 1 alongside its chemical structure. dI/dV spectra were recorded at the positions marked by red, blue, green, purple, yellow, and light blue dots in its BR-STM image, along with a reference spectrum from Au(111). The d^2I/dV^2 spectrum at the red-marked position is recorded. (b) BR-STM image of dimer 2 alongside its chemical structure. dI/dV spectra were recorded at the positions marked by red, blue, green, purple, and light blue dots in its BR-STM image, with a reference spectrum from Au(111) included. The d^2I/dV^2 spectrum at the red-marked position is recorded. Measurement parameters: BR-STM images: $V = 1$ mV, $V_{mod} = 10$ mV. dI/dV and d^2I/dV^2 spectra: $V = 100$ mV, $I = 200$ pA, $V_{mod} = 2$ mV.

It is worth noting that the observed spin excitation energies in the above dimers (Figure S6a and S6b) are distinct significantly. While one would generally expect that a longer distance between radical units leads to a weaker magnetic coupling, the relatively high exchange interaction in Figure S6b can arise from several factors beyond the distance.

First, and most importantly, the exchange interactions can be mediated by the RKKY-like interaction of the nanographene units on the metal substrate, allowing indirect magnetic interaction via conduction electrons, that can easily reach tens of meV at nanometer distances and behavior depending on the sublattices where the magnetic moments are located. Furthermore, as demonstrated, tip-induced dehydrogenation can result in chemical bonding between the quenched unit and the Au substrate, which can alter the local density of states and facilitates substrate-mediated hybridization. These effects can enhance the effective exchange interactions, particularly in geometries where direct radical-radical overlap is reduced. In addition, in spin rings there are typically two competing interactions, ferromagnetic and antiferromagnetic. In the hexamer and pentamer, ferro- and antiferromagnetic interactions coexist along different paths, leading to frustration and partial cancellation of the net interaction strength. In the half-quenched hexamer, this competition might be reduced, and antiferromagnetic tendency can be more effectively preserved. As a result, the stronger antiferromagnetic interactions contribute more to the overall exchange coupling.

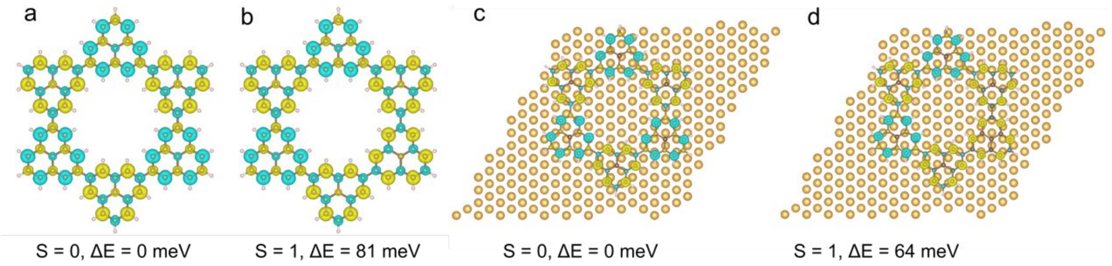

Figure S7. Calculated spin density distributions and corresponding energy difference of [2]triangulene hexamer ring with different spin configurations. (**a, b**) Freestanding hexamer ring with a singlet state (S = 0) and a triplet state S = 1, respectively. (**c, d**) [2]Triangulene hexamer ring on Au(111) in a singlet state (S = 0) and a triplet state (S = 1), respectively. The total energy of the singlet state is set as zero. Both the calculations for the freestanding hexamer and the hexamer on Au(111) reveal that the antiferromagnetically coupled singlet state is energetically favored.

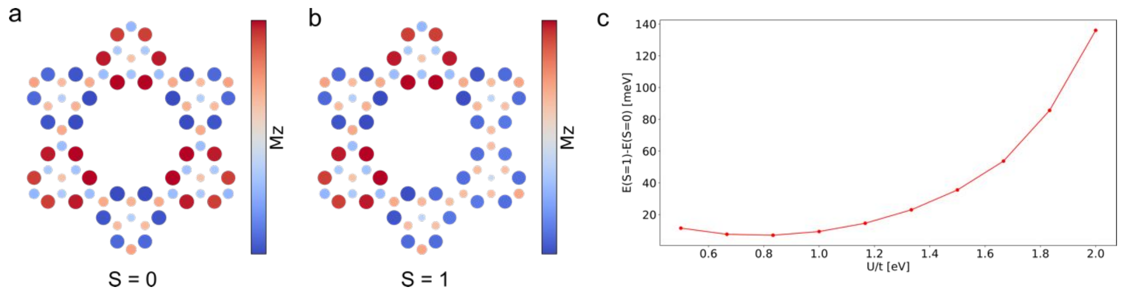

Figure S8. Mean-field Hubbard calculations of [2]triangulene hexamer ring. (**a, b**) Hexamer ring with an antiferromagnetic singlet state (S = 0) and an excited S = 1 state, respectively. (**c**) Energy difference between the excited triplet state (S = 1) and singlet ground state (S = 0) of the hexamer ring plotted as a function of U/t.

We investigated the magnetic properties of [2]triangulene hexamer ring by MFH calculations. The MFH Hamiltonian for the carbon p_z orbitals includes only the nearest neighbor hopping term (t=2.7 eV) as well as the onsite Coulomb repulsion U within the mean-field approximation. The calculated spin density distributions of the singlet ground and excited triplet states align with those calculated by spin-polarized DFT. The simulations predict the energetically favored antiferromagnetic singlet ground state of the [2]triangulene hexamer ring, aligning our spin-polarized DFT calculations.

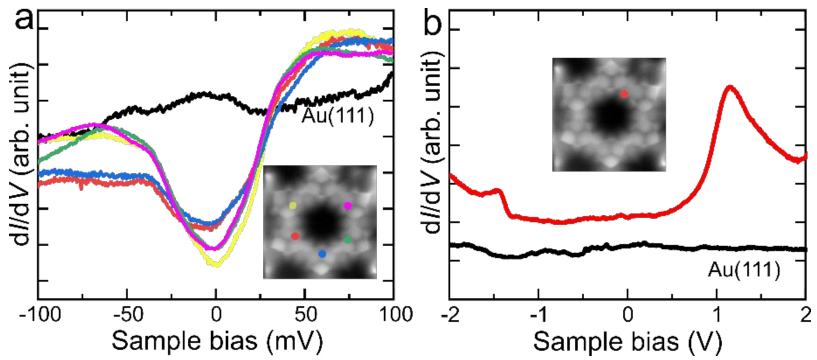

Figure S9. d*I*/d*V* spectra of [6]TSR. (**a**) Low-energy d*I*/d*V* spectra taken at the positions marked by black, red, blue, green and pink dots in the inset. Black curve was taken over the bare Au(111) surface. Lock-in amplitude: V_{mod} =2 mV. (**b**) Long-range d*I*/d*V* spectra taken at the position marked by red dot in the inset. Black curve was taken over the bare Au(111) surface. Lock-in amplitude: V_{mod} = 10 mV.

We performed long-range scanning tunneling spectroscopy (STS) measurements to reveal the electronic properties of hexamer. As shown in fig. S9b, we recorded the characteristic differential conductance spectra over the bridge (red curve) of the hexamer, as well as the spectrum collected on the bare Au(111) surface (black curve). These d*I*/d*V* spectra show several peaks at bias voltages of −1.5 V, −0.5 V, and a broad peak from 1.0 V to 1.6 V.

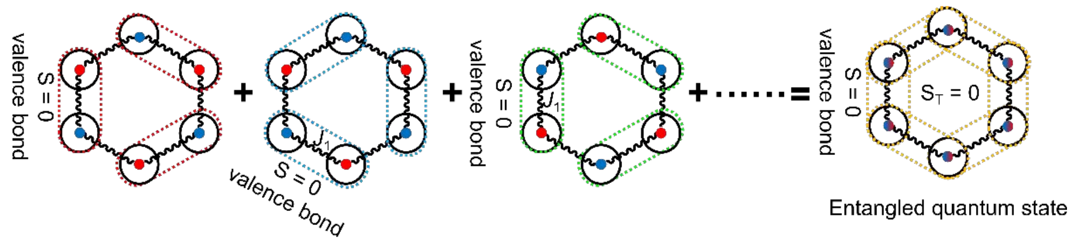

Figure S10. Schematic illustration of the formation of the resonating valence-bond state. Due to the quantum fluctuations, the spins would constantly alter their singlet partners and rearrange the pairings. Therefore, a many-body analogue of the spin-singlet ground state, the so-called resonating valence bond (RVB) state, was formed by superimposing different spin states at each site to stabilize the system. Red and blue filled circles denote spin up and spin down electrons, respectively.

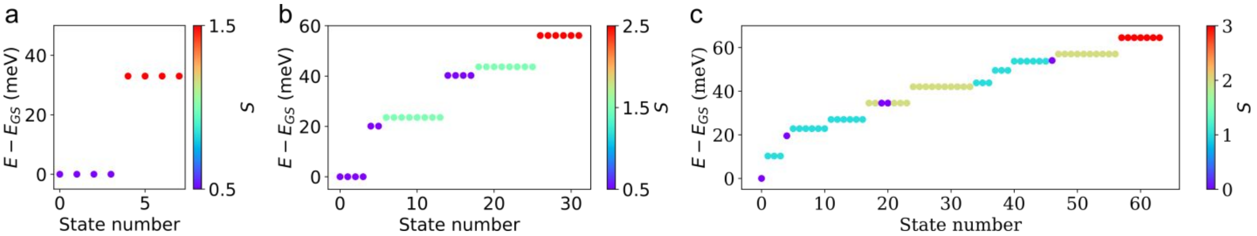

Figure S11. Energy diagrams of spin excitation states obtained from the Heisenberg model. (a) Half-quenched hexamer computed with J = 22 meV, (b) Pentamer with J = 18 meV, and (c) Hexamer with J = 15 meV. The color scale indicates the total quantum spin number S_T of each state, and the number of dots at a given energy level represents the degeneracy of that state. For both pentamer and half-quenched hexamer, a four-fold degenerate ground state with S_T = 1/2 is observed. The half-quenched hexamer exhibits a first excitation state at 33 meV with four-fold degeneracy and S_T = 3/2, consistence with the excitation signal observed in the experimental d^2I/dV^2 spectra. In the pentamer, the first excited state is a doublet with S_T = 1/2 at 20 meV, followed closely by a second excitation state at 23 meV. These features correspond to experimentally observed excitation at 24 meV. The computed energy diagram of the hexamer shows a singlet ground state, followed by a triple first excited state with S_T = 1 at 10 meV. The second excited state is a singlet (S_T = 0) at 23 meV, closely followed by a six-fold degenerate state at 27 meV. These two states correspond to the experimental resolved excitation at 25 meV. A triplet state with S_T = 1 at 49 meV align with the experimentally observed excitation at 48 meV.

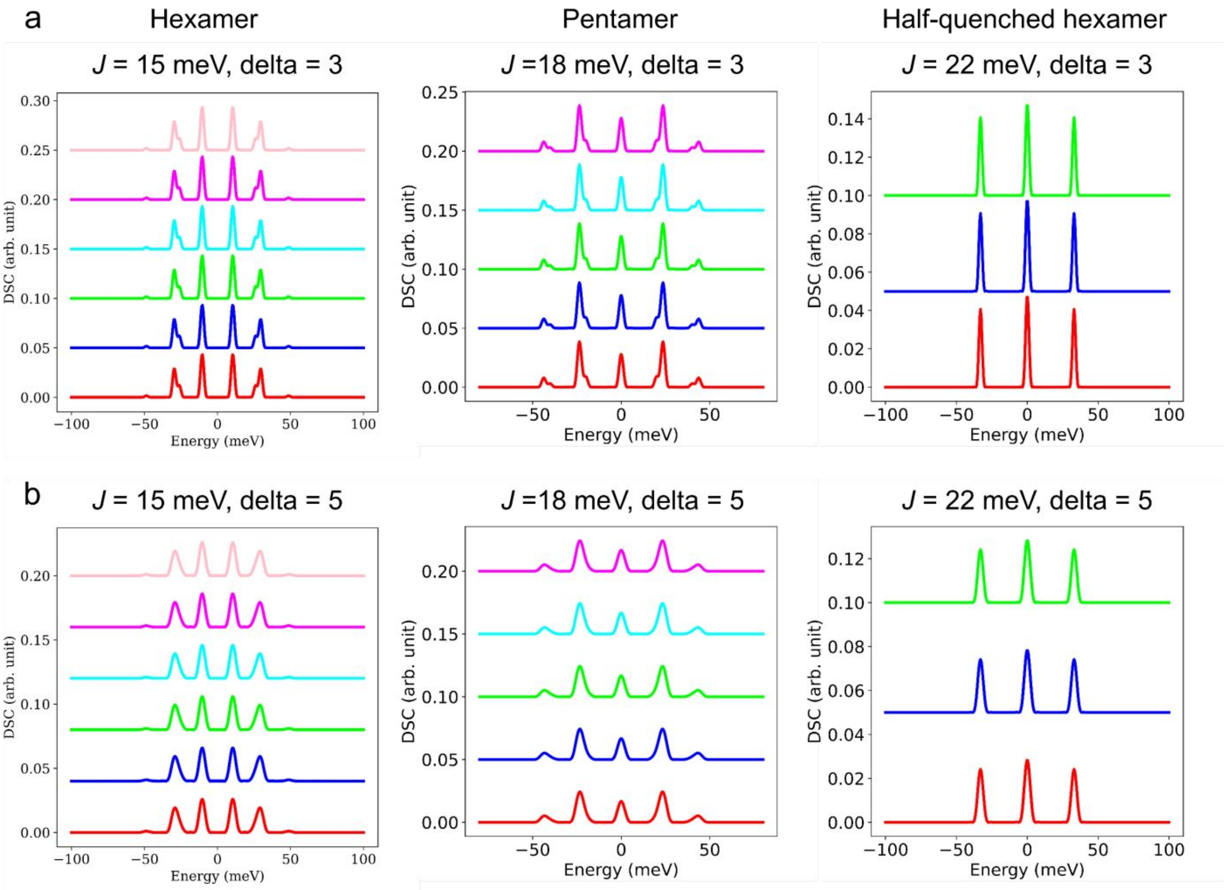

Figure S12. Computed full dynamic spin correlators for each unit of the hexamer, pentamer, and half-quenched hexamer spin rings. The calculations are performed using nearest neighbor exchange coupling J, with delta function broadening parameters of 3 in (a) and 5 in (b), illustrating broadening effects to the spectra. The color of each curve represents the DSC spectra computed on each unit. Excitation energies are consistent across all units within each ring. Notably the pentamer and half-quenched hexamer exhibit zero-bias signals, indicating a total spin $S_T = 1/2$ ground state in both spin rings.

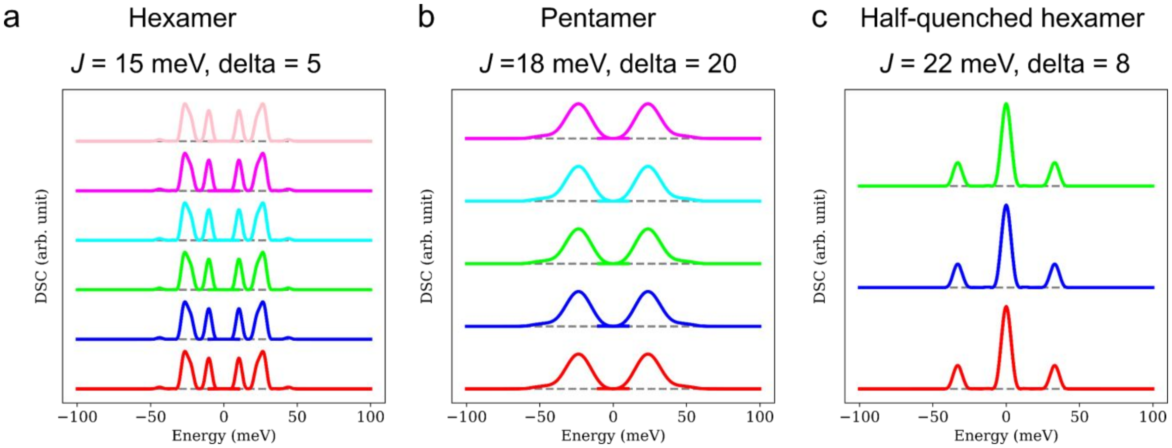

Figure S13. Computed dynamic spin correlators showing local spin-flip excitation spectra at site n for singly degenerate ground state of spin rings. The calculations are performed using nearest neighbor exchange coupling *J*, with delta function broadening parameters specified in the (a) the hexamer, (b) the pentamer, and (c) the half-quenched hexamer. The color of each curve represents the DSC spectra computed on each unit. The dominate excitation energies are consistent with those obtained using the full correlator.

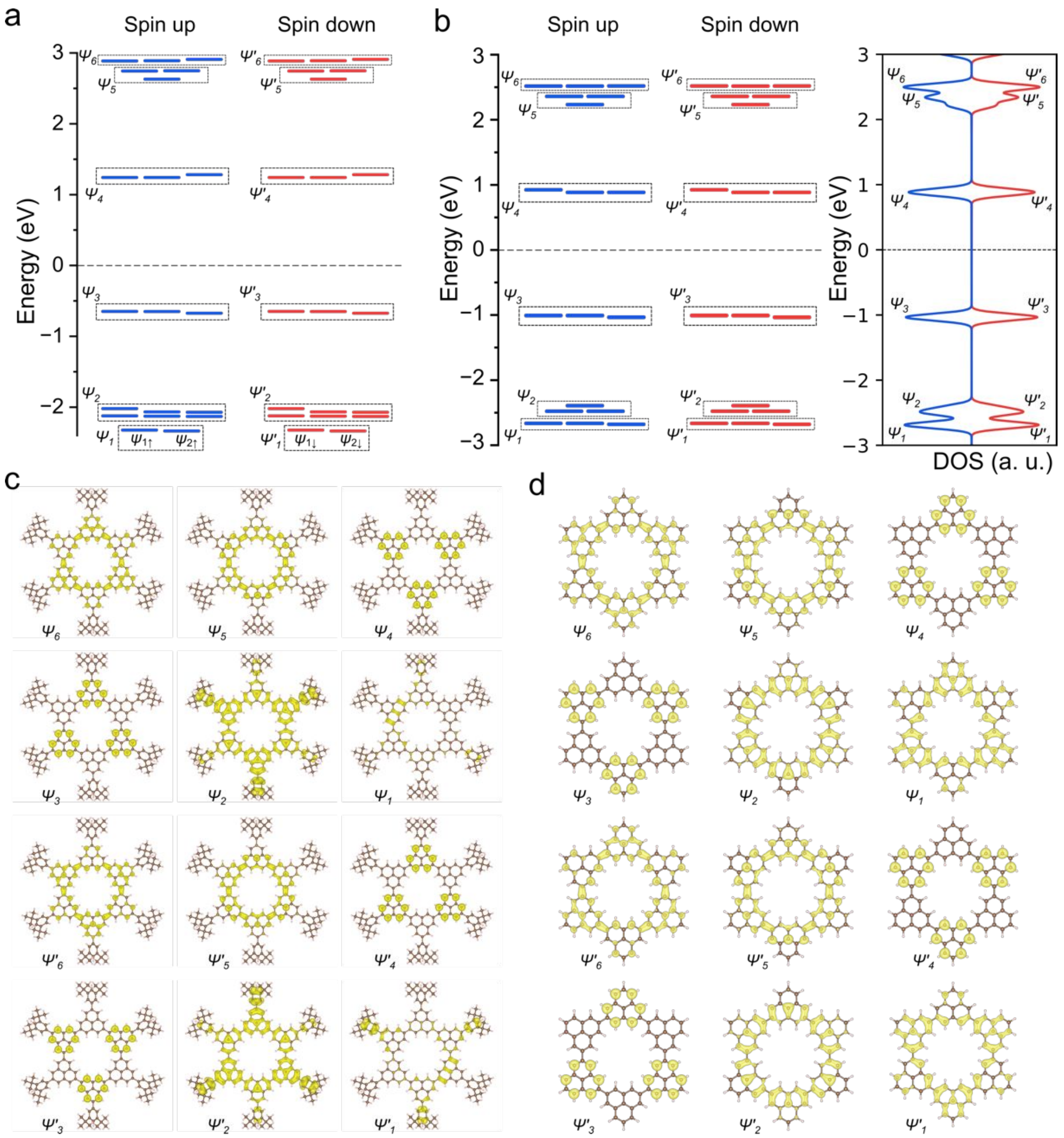

Figure S14. Spin-polarized calculations of energy levels and orbital densities of frontier molecular orbitals for the hexamer with bulky groups and the [2]triangulene hexamer ring. (**a, c**) depict the energy level diagrams and the corresponding molecular orbital plots of the hexamer with bulky groups, respectively. (**b, d**) The calculated energy levels and density of state and molecular orbital plots for the [2]triangulene hexamer ring , respectively. The orbital densities for each state Ψ are described as the combination of orbitals that are close in energy (indicated with dashed rectangles). For example, $\Psi_1 = |\psi_{1\uparrow}|^2 + |\psi_{2\uparrow}|^2$ and $\Psi'_1 = |\psi_{1\downarrow}|^2 + |\psi_{2\downarrow}|^2$. Comparison of these results indicates that the bulky groups have only a minor contribution to the electronic distribution of the molecule in the energy range of −3 to 3 eV.

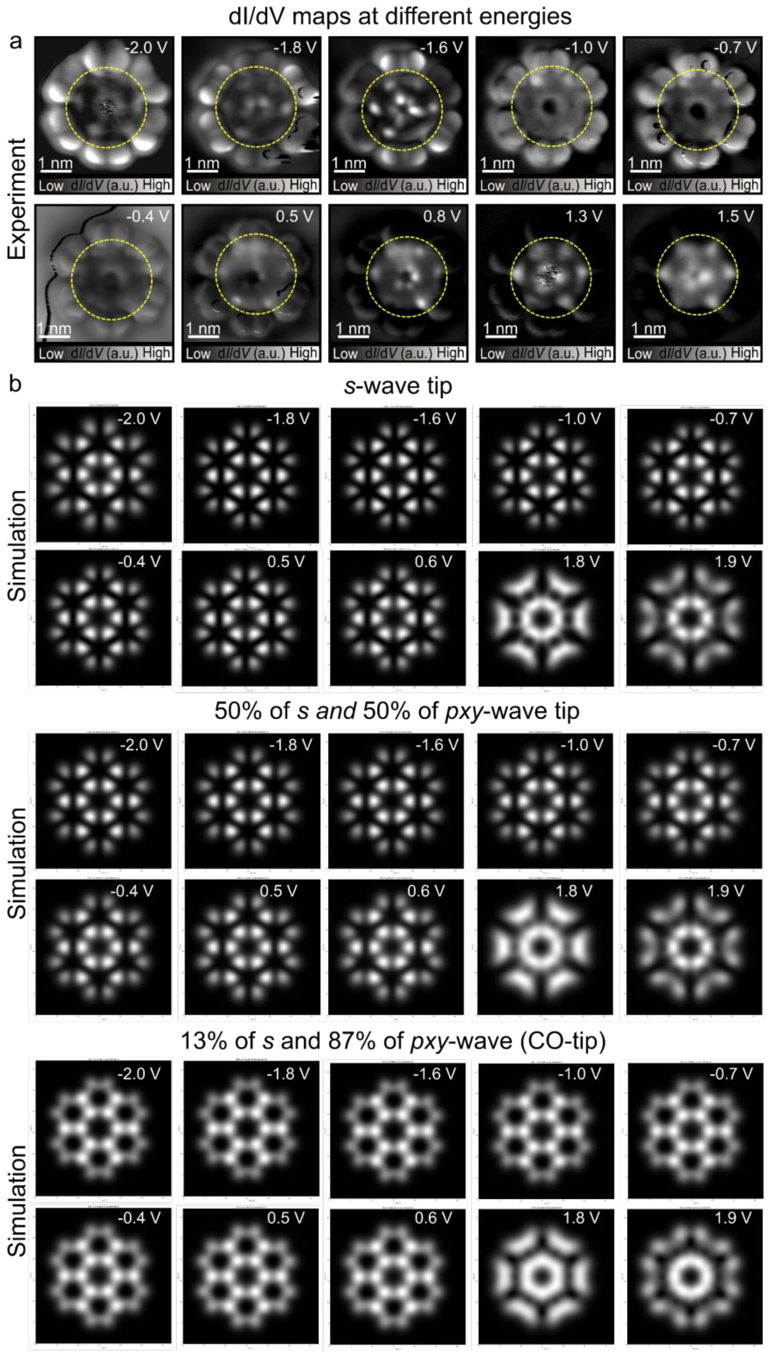

Figure S15. Experimental and simulated d*I*/d*V* maps of the cyclic hexamer. (**a**) Experimental d*I*/d*V* maps taken on the intact cyclic hexamer at constant-height mode using CO-tips. (**b**) Constant-height d*I*/d*V* map simulations of the triangulene ring using various tip models, corresponding to the inner structures in (a), the region shown by the dashed yellow circles in (a). The simulations reproduce the main features of experimentally observed electronic distributions in a broad energy range. Each simulated map has a size of 3.2×3.2 nm^2.

To reproduce the experimental contrast, we tested various tip models with different orbital compositions, pure *s*-wave, and mixed *s* and *pxy* waves. While an *ideal* CO-functionalized tip consists of 13% *s* and 87% *pxy* waves, simulations using a pure *s*-wave tip or 50% s + 50% *pxy*-wave tip give rise to spatial features that more closely resemble the experimental maps. Notably,

increasing the *s*-wave component in the tip enhances the agreement with the experiment. Although experimental data were acquired using CO-tips, the effective tunneling symmetry can vary due to differences in tip apex structure or tilt angle, which may result in a more *s*- or *sp*-like character. Nevertheless, using these different tip models does not affect the interpretation of the electronic or magnetic states, and the qualitative agreement between the simulation and experiment remains robust.

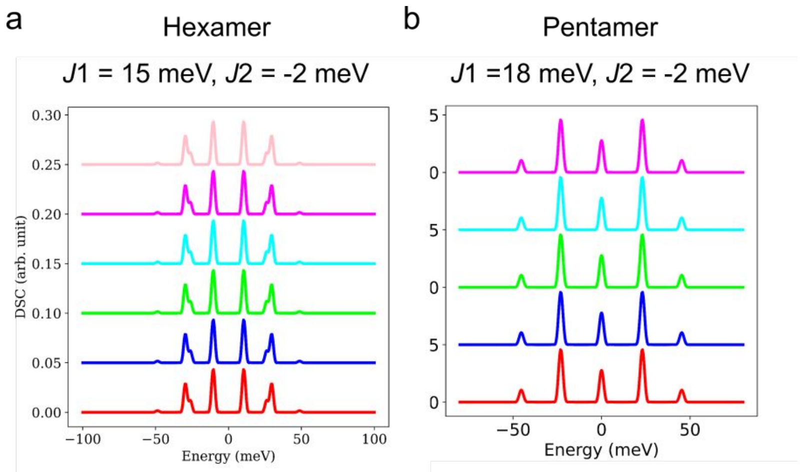

Figure S16. Computed full dynamic spin correlators for (a) the hexamer and (b) the pentamer, including both nearest neighbor ($J1$) and next-nearest neighbor ($J2$) spin interactions, with $J2 \approx 0.1\times J1$. Compared to the DSC spectra obtained with only $J1$ (Figure S17), the inclusion of $J2$ leads to a slight downward shift in excitation energies. While the overall spectral features and magnetic behavior remain largely unchanged, indicating that the spin systems are still primarily dominated by the nearest-neighbor coupling.

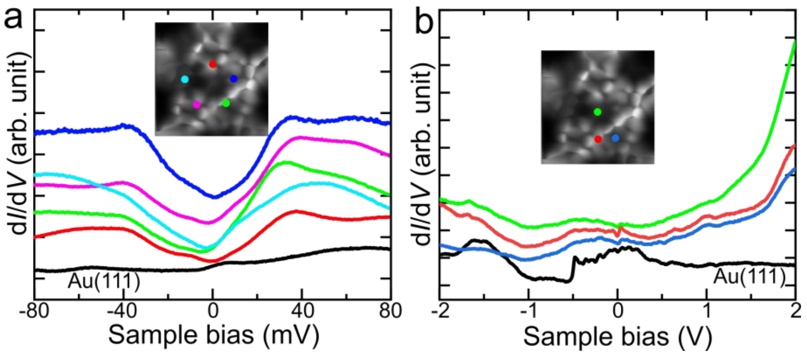

Figure S17. dI/dV spectra of [5]TSR. (**a**) Low-energy dI/dV spectra taken at the positions marked by red, blue, light blue, purple, and green dots in the inset. Black curve was taken over the bare Au(111) surface. Lock-in amplitude: V_{mod} = 2 mV. (**b**) Long-range dI/dV spectra taken at the position marked by red and blue dots in the inset. Black curve was taken over the bare Au(111) surface. Lock-in amplitude: V_{mod} = 10 mV.

As shown in Figure S17a, Figure 3d and 3h, the spin excitations vary across the pentamer ring, whereas the theoretical computations in Figure 3f and Figure S12 exhibit identical spectra at each unit. This discrepancy is due to the spin model used in our calculations. Specifically, we use a symmetric Heisenberg Hamiltonian with uniform exchange couplings and isotropic interactions, which assumes perfect structural and electronic symmetry for all units. Under these assumptions, the excitation spectra are expected to be identical at each unit. However, experimental systems inevitably deviate from this theoretical model. Potential factors include: (i) Nonplanar geometry and local structural distortions within the pentamer can lead to differences in the overlap between local spins and the STM tip, affecting the spectral intensity and energy at each unit. (ii) Substrate-induced hybridization, which can vary depending on the adsorption configuration of each unit, resulting in site-dependent electronic environments and further modify the local excitation energies. These effects can break the local symmetry and are not captured by our spin Hamiltonian. Nevertheless, our model accurately reproduces the main spin excitations.

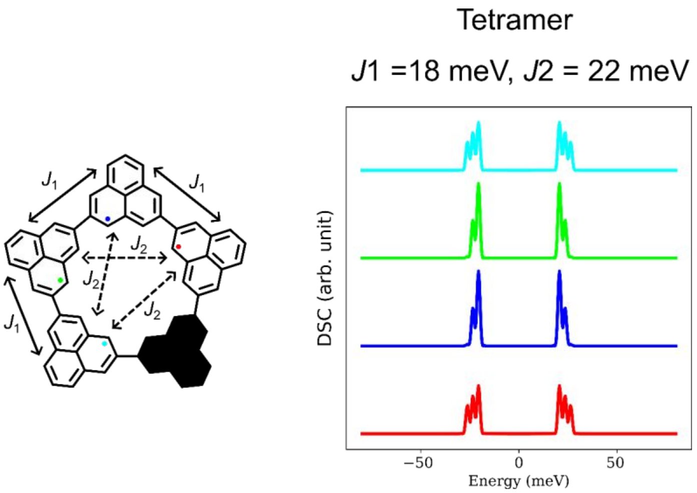

Figure S18. Full dynamical spin correlator of an asymmetric tetramer spin ring. As shown in Figure S17, although one of the units in the pentamer may experience stronger hybridization due to its local adsorption configuration, the d*I*/d*V* spectra exhibit nearly identical dip features between five units. This suggests that the spins remain on each unit. To evaluate the possibility that one of the units is quenched in the pentamer, i.e., the scenario of an asymmetric tetramer, we computed DSC spectra for an asymmetric tetramer ring with three nearest neighbor (J_1 = 18 meV) interactions and three next-nearest neighbor (J_2 = 22 meV) interactions, as shown in the scheme. The J_2 value was chosen to match that of the half-quenched hexamer, because of their comparable distance between next-nearest neighbor units. The DSC spectra shows significant variations in excitation spectral between units. This is not observed in our experiment. We therefore conclude that the asymmetric tetramer model does not accurately describe the experimental pentamer and we rule out this possibility.

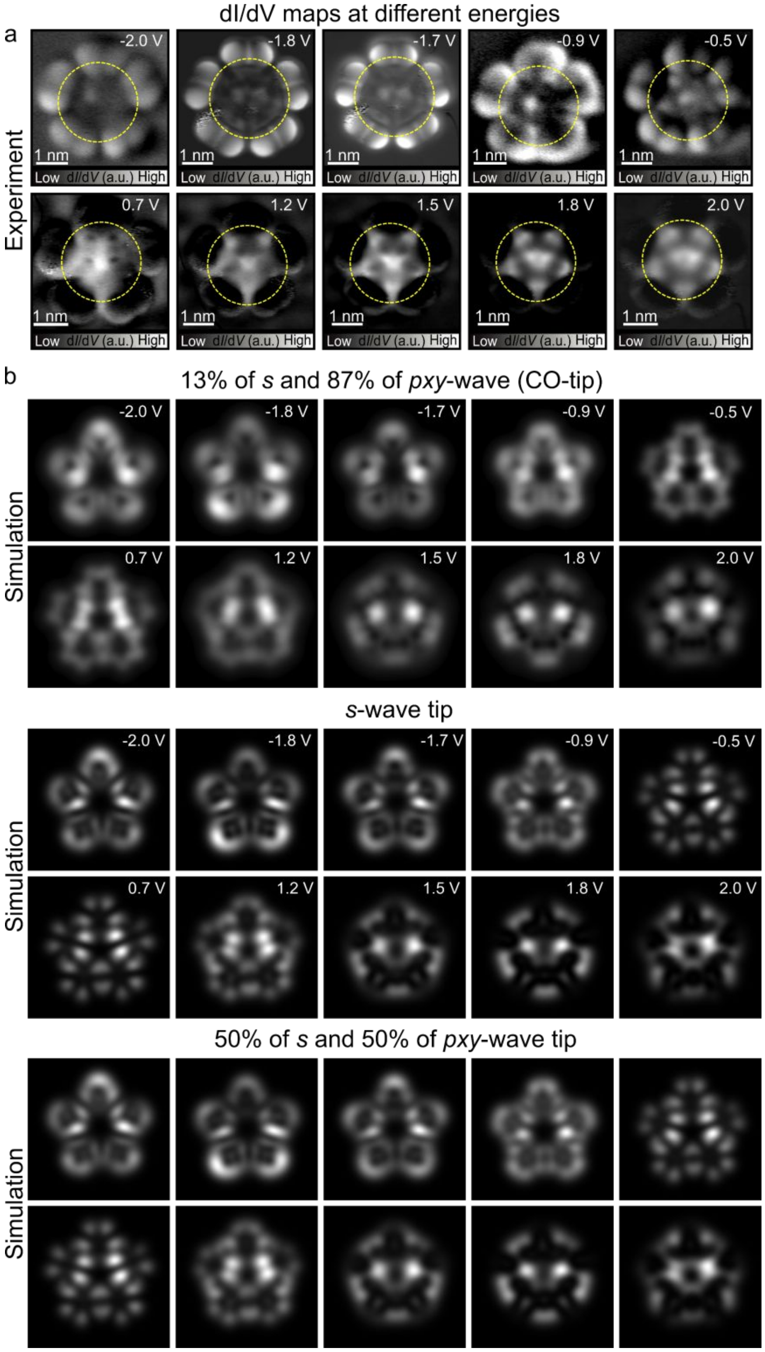

Figure S19. Experimental and simulated dI/dV maps of the cyclic pentamer. (**a**) Constant-current dI/dV maps taken on the intact cyclic pentamer using CO-tips. (**b**) Constant-height dI/dV map simulations of the cyclic triangulene ring based on the Structure 8 searched by BOSS (Figure S3), corresponding to the inner structures indicated by dashed yellow circles in (a). Similar to the simulations for hexamer, we also used various tip models to assess the influence of realistic tunneling. Overall, the simulations reproduce the main experimental features in the positive energy range. While in the negative energy range, the simulations using an *ideal* CO-functionalized tip (13% *s* and 87% *pxy* waves) are more resemble the experimental maps.

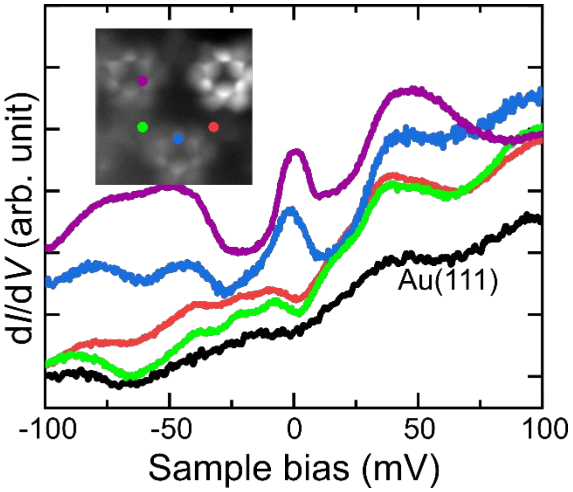

Figure S20. Magnetic and electronic properties of half-quenched hexamer. Low-energy d*I*/d*V* spectra taken at the positions marked by black, red, blue, green and blue dots in the inset. Black curve was taken over the bare Au(111) surface. Lock-in amplitude: 2 mV.

Note S1. Heisenberg Hamiltonian. We employed exact diagonalization to calculate the eigenenergies and eigenstates of spin rings with Heisenberg Hamiltonian. The spin systems consist of $S = 1/2$ spins of size N that are coupled antiferromagnetically with exchange coupling J to each two other spins to model the system in a closed ring. The exchange constant J was tuned to fit the excitation energies in the experiment, we used $J = 24$ meV for $N = 3$, $J = 18$ meV for $N = 5$, 7, and $J = 15$ meV for $N = 6$.

$$\hat{H} = J\left(\sum_{i=0}^{N-2} S_i \cdot S_{i+1} + S_{N-1} \cdot S_0\right)$$

Note S2. Origin of the ground state degeneracy. The four-fold degenerated ground states of the [2]triangulene pentamer ring arise from the interplay between the geometric symmetry of the system and the spin symmetry of spin-1/2 particles.

The spin Hamiltonian conserves the total spin projection $S_z = \pm\ 0.5$ in the ground state. Geometrically, the pentamer exhibits five-fold cyclic rotational symmetry (C_5), corresponding to the translation symmetry of the Hamiltonian. With closed boundary conditions, the spin Hamiltonian H commutes with the translation operator T,

$$H|\Psi\rangle = E|\Psi\rangle,$$
$$[H,T] = 0$$

It means that translating the spin between neighboring sites does not change the Hamiltonian (cyclically permuted), meaning the energy of the system remains unchanged, and H and T share the same eigenstates. Therefore, the eigenstates of H (energy states) can be labeled by the eigenvalues of translation operator T,

$$T|\Psi\rangle = e^{i\phi}|\Psi\rangle$$

The translation operator T shifts the spin states in the ring by one site. In a pentamer, performing 5 translations brings the system back to its original configuration. The operator T satisfies:

$$T^5 = I \text{ (identity operator) } \Rightarrow\ e^{i\phi 5} = 1$$

where $e^{i\phi}$ is a complex number representing the geometric phases under translations by $2\pi/5$, determined by the momentum quantum number n. The allowed eigenvalues of T are:

$$e^{i\phi} = e^{i2\pi n/5}, n = 0, 1, 2, 3, 4$$

Possible phases of the wavefunction are:

$$\phi = \pm \frac{2\pi n}{5}, n = 0, 1, 2, 3, 4$$

Due to the symmetry properties of the ring, the phase can be expressed as:

$$\phi = 0,\ \pm\frac{2\pi}{5},\ \pm\frac{4\pi}{5}$$

The spin wavefunctions can be acquired either positive or negative phase with a rotation by $2\pi/5$. The numerically determined ground state manifold corresponds to momentum sectors $n = 1$, and $n = 4$, corresponding to translation phases $\phi = \pm \frac{2\pi}{5}$. These sectors are conjugates of one another, as $e^{-i2\pi/5}$ is equivalent to $e^{i2\pi \cdot 4/5}$, and represent winding clockwise and anticlockwise around the ring. To label them, we introduce an internal quantum number C, where $C = +1$, denotes $\phi = +\frac{2\pi}{5}$ and $C = -1$, denotes $\phi = -\frac{2\pi}{5}$. Therefore, for each $S_z = \pm\ 0.5$, there are two distinct winding configurations ($C = \pm 1$), resulting in four degenerate ground states. This discussion can be directly applied to the trimer, leading in that case also to a four-fold degenerate ground state.

In stark contrast, for even-numbered rings like the hexamer do not support such winding-induced degeneracy, which corresponds to having a ground state with C=0. We found that the ground state of the hexamer is a singlet with total spin S_T=0, in which all spins are paired into singlets. This state belongs to the trivial momentum sector $n = 0$ and is invariant under cyclic translation. Since no unpaired spin exists to generate distinct momentum eigenstates, and the singlet is symmetric under rotation, the ground state is singly degenerate. Although translation symmetry is still present, it acts trivially on the singlet, and the internal quantum number C corresponds to C=0

Note S3. Dynamical spin correlator. In this note, we provide a theoretical discussion and interpretation of the dynamical spin correlator (DSC) used to compute the spin excitations in finite quantum spin ring systems.

Local dynamical spin correlator. We computed the local spin-flip excitation spectra at site *n for a singly degenerate ground state*, the spectral function is:

$$\boldsymbol{A}(\omega,n) = \langle GS|S_n^- \delta(\omega + E_{GS} - \hat{H}) S_n^+|GS\rangle$$

where $|GS\rangle$ and E_{GS} refer to the many-body ground state and the energy obtained by exact diagonalization of the Heisenberg Hamiltonian $\hat{H}$ with exchange coupling J. S_n^- and S_n^+ are spin operators for lowering and raising a spin and $S_n^{\pm} = S_n^x \pm iS_n^y$. $\delta(\omega + E_{GS} - \hat{H})$ is an operator valued delta function that resolves transitions at frequency ω. This function captures the energy resolved spin flip excitation at site n. In the context of inelastic electron tunneling spectra, the frequency of the excitation ω corresponds to the applied bias voltage in the experiment, n indicates the unit that is treated in the ring.

In the presence of ground state degeneracy, a specific ground state needs to be selected to apply the formulation above. In the presence of spin and cyclic symmetry, the choice of ground state would need to be carefully done so that the dynamical correlators do not break the symmetries of the problem. A minimal way of selecting a ground state with the right symmetries is by applying a very small spiral magnetic field in the *xy*-plane to select one of the states of the ground state manifold. The magnetic field is defined as,

$$\vec{B}_i = B_z(cos\,\theta_i, sin\,\theta_i, 1), \theta_i = \frac{2\pi i}{N},$$

By computing $\boldsymbol{A}(\omega)$ for all sites n, we obtain a spatially resolved dynamical correlator.

$$\boldsymbol{A}(\omega,n) = \sum_m |\langle m|S_n^+|GS\rangle|^2 \delta(\omega + E_{GS} - E_m)$$

where $|m\rangle$ are eigenstates of the Hamiltonian with energy E_m. The matrix element $\langle m|S_n^+|GS\rangle$ reflects how strongly the spin operator couples the ground state to each excited state. Such matrix element depends on the exact form of the coupling between the STM and the ring. In particular, if the STM is coupled to different sites of the ring simultaneously, the dynamical correlator can be redefined with the neighboring spins $S_n^+ + S_{n+1}^+$, resulting in the expression:

$$\boldsymbol{A}''(\omega,n) = \langle GS|(S_n^- + S_{n+1}^-)\delta(\omega + E_{GS} - \hat{H})(S_n^+ + S_{n+1}^+)|GS\rangle$$

This operator introduces the nearest neighbor contribution to the spin operator, which allows better account for potential coupling to several sites simultaneously.

Full dynamic spin correlator. To obtain a full spin excitation spectrum, including contributions from all spin directions and all ground states in a degenerate manifold, we compute the full dynamical correlator,

$$\boldsymbol{S}_{ij}^{full}(\omega) = \frac{1}{3M}\sum_{m=1}^{M}\sum_{\alpha=x,y,z}\left\langle \varphi_{GS}{}^{m}|S_i^{\alpha}\delta(\omega + E_{GS} - \hat{H})S_j^{\alpha}|\varphi_{GS}{}^{m}\right\rangle$$

where $|\varphi_{GS}{}^{m}\rangle$ are all M degenerate ground states, and α runs over all spin directions. In our implementation, the full correlator is computed by looping over each ground state wavefunctions and evaluating the correlator for each spin component. The resulting spectra are averaged over both ground state manifold and spin directions.

This approach captures all energetically allowed spin excitations, including those that may be invisible in single ground state calculation due to selection rules. It is especially important for systems with ground state degeneracies, such as the geometrically frustrated N=3,5 closed rings where different symmetry sectors may host distinct excitations. In the case of a singly degenerate ground state, the full correlator becomes equivalent to the single dynamical correlator described in the previous section.

Delta function. The delta function in the dynamic spin correlator is approximated numerically using the kernel polynomial method (KPM) via a Chebyshev polynomial expansion. Before the expansion, the original Hamiltonian $\hat{H}$ is linearly rescaled to fit the interval [-1, 1], required by the KPM method. To compute the dynamic correlator, we take the form after an expansion:

$$\overline{\boldsymbol{S}}(\omega) = \frac{1}{\pi\sqrt{1-\omega^2}}\left[\mu_0 + 2\sum_{l=1}^{N_p} g_l^{N_p}\mu_l T_l(\omega)\right]$$

Where $T_l(\omega$ are Chebyshev polynomials and $\mu_l = \langle GS|S_i^z T_l(\overline{H})S_i^z|GS\rangle$ are the expansion coefficients. $g_l^{N_p}$ are the Jackson Kernel used to smooth Gibbs oscillations. This approximation introduces a controlled spectral broadening scales as $1/N_p$ over the rescaled domain. The apparent broadening in the discrete excitation spectrum in the DSC spectra results from the numerical representation of the delta function. The degree of smoothing can be tuned via N_p, the bigger the number of polynomial N_p the sharper the spectral feature will be.

In our implementation, the user-defined delta parameter serves as a practical input to specify the desired spectral resolution. It is internally related to the number of Chebyshev polynomials N_p via the relation $\delta = C \times \frac{\text{bandwidth}}{N_\text{p}}$, where C is a prefactor determined by the specific implementation (with C = 0.5 in our case), and bandwidth refers to the energy range of the rescaled Hamiltonian. Thus, the delta parameter used in our DSC calculations is dimensionless within the rescaled domain, but corresponds to a physical energy resolution when mapped back to the original energy scale.

In-solution synthesis of precursor radical 1

All reactions involving air- or moisture-sensitive reagents were carried out using standard Schlenk line and glove box techniques using heat-gun dried glassware. Unless noted otherwise the work-up was performed under ambient conditions. Dehydrated solvents were purchased from Kanto Chemical Co., Inc. and used without further purification. 2-(3,5-di-*tert*-butylphenyl)-2,3-dihydro-1*H*-phenalen-1-one (**2**) was synthesised according to previous literature procedures (*42*). Column chromatography was performed using silica gel 60N (Kanto Chemical Co., Inc.).

Scheme S1. Synthetic route 5,8-dibromo-2-(3,5-di-*tert*-butylphenyl)-[2]triangulene [a]

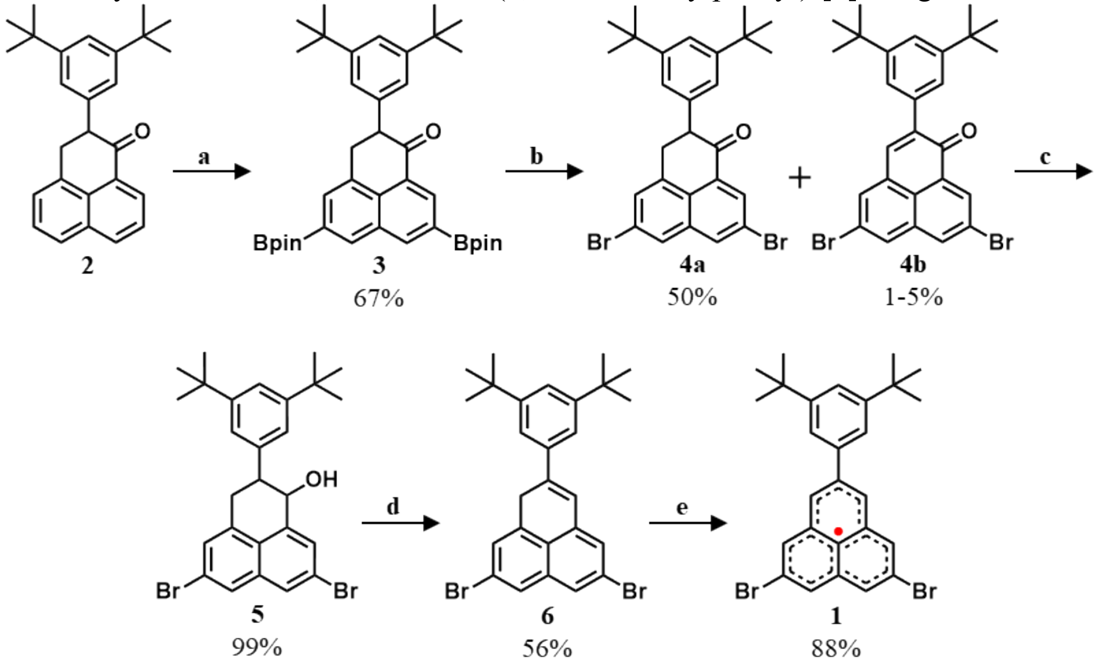

[a] Reaction conditions: (a) bis(pinacolato)diboron, 4,4'-di-*tert*-butyl-2,2'-bipyridyl, bis(1,5-cyclooctadiene)di-μ-methoxydiiridium(I), tetrahydrofuran, 80 °C, 16 h; (b) $CuBr_2$, *N*,*N*-dimethylformamide, water, methanol, 80 °C, 48 h; (c) $NaBH_4$, $CeCl_3 \cdot 7H_2O$, methanol, dichloromethane, 0 °C to rt, 2h (d) *p*-toluenesulfonic acid monohydrate, toluene, reflux, 16 h; (e) *p*-chloranil, toluene, 50 °C, 16 h.

5,8-bis(Bpin)-2-(3,5-di-*tert*-butylphenyl)-2,3-dihydro-1*H*-phenalen-1-one (**3**)

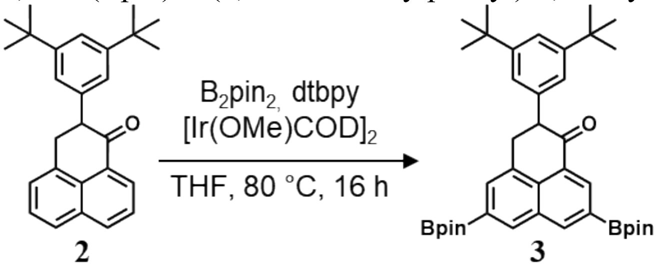

2 **3**
67%

2-(3,5-di-*tert*-butylphenyl)-2,3-dihydro-1*H*-phenalen-1-one (**2**, 2.000 g, 5.4 mmol, 1.0 eq.), 4,4'-di-*tert*-butyl-2,2'-bipyridyl (0.087 g, 0.32 mmol, 0.06 eq.), bis(1,5-cyclooctadiene)di-μ-methoxydiiridium(I) (0.107 g, 0.162 mmol, 0.03 eq.), and bis(pinacolato)diboron (3.427 g, 13.5 mmol, 2.5 eq.) were added to a dry Schlenk bomb flask under Ar. Anhydrous and degassed THF (~50 mL) was added to the flask that was sealed under Ar and heated to 80 °C under stirring for 16 h. The cooled reaction mixture was diluted with water and dichloromethane. The organic phase was separated and washed with water and brine. The combined organic phase was dried over Na_2SO_4 and the solvent evaporated. The product mixture was filtered through a short silica plug (eluent: dichloromethane) and further purified by column chromatography (silica gel; eluent: dichloromethane) to receive the product **3** as yellow-orange solid (2.399 g, 67%). **^{1}H-NMR** (400 MHz, $CDCl_3$): δ_H = 8.62–8.61 (m, 2H; Ar-H), 8.37 (d, *J*=1.1 Hz, 1H; Ar-H), 7.90 (d, *J*=1.1 Hz, 1H; Ar-H), 7.27 (t, *J*=1.8 Hz, 1H; Ar-H), 7.08 (d, *J*=1.8 Hz, 2H; Ar-H), 4.21 (t, *J*=7.2 Hz, 2H), 3.74 (d, *J*=7.2 Hz, 2H), 1.40 (s, 12H, Bpin), 1.37 (s, 12H, Bpin), 1.23 (s, 18H, *t*-Bu) ppm. **^{13}C-NMR** (100 MHz, $CDCl_3$): δ_C = 198.38, 150.70, 142.58, 137.74, 135.43, 134.42, 132.17, 132.09, 132.06, 131.20, 128.98, 127.02 (broad, C-B), 122.76, 121.25, 84.32, 84.23, 53.83, 35.83, 34.94, 31.54, 25.17, 25.11, 25.07, 25.03, 24.98 ppm. **HRMS (APCI)**: *m*/*z* calcd. for $C_{39}H_{52}B_2O_5$ $[M+H]^+$ 623.4087, found 623.4088.

5,8-dibromo-2-(3,5-di-*tert*-butylphenyl)-2,3-dihydro-1*H*-phenalen-1-one (**4a**)

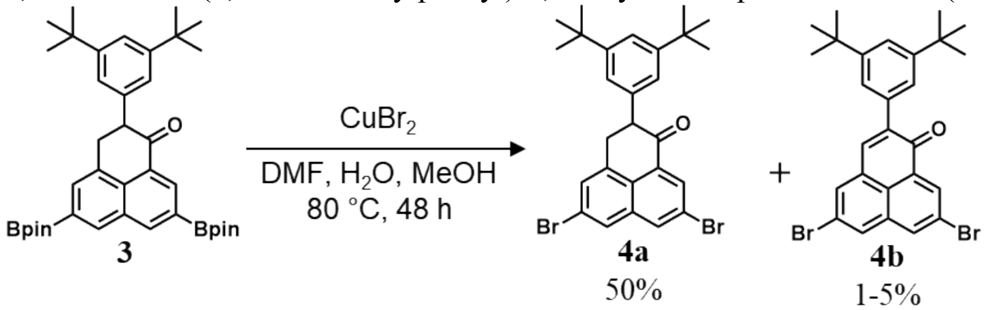

3 **4a** 50% **4b** 1-5%

Compound **3** (2.000 g, 3.2 mmol, 1.0 eq.) was added to a Schlenk flask and dissolved in a mixture of thoroughly degassed methanol (120 mL) and *N*,*N*-dimethylformamide (40 mL). In a separate Schlenk flask $CuBr_2$ (4.306 g, 19.3 mmol, 6.0 eq.) was dissolved in degassed water (120 mL), and cannulated to the reaction flask under strong stirring. The reaction mixture was stirred under Ar for 48 h at 80 °C. The product mixture was cooled with an ice bath, diluted with water, and extracted with dichloromethane. The separated organic phase was washed with water and brine, dried over $MgSO_4$, and the solvent was evaporated under vacuum. Purification *via* column chromatography (silica gel; eluent: *n*-hexane/dichloromethane (2:1 v/v)) gave **4a** as yellow solid (0.852 g, 50%) with varying amounts (1–5%) of oxidized side product **4b**.

4a: ^{1}**H-NMR (400 MHz, CDCl$_3$)**: δ_H = 8.25 (d, *J*=2.0 Hz, 1H; Ar-H), 8.13 (d, *J*=2.0 Hz, 1H; Ar-H), 7.90 (d, *J*=1.9 Hz, 1H; Ar-H), 7.62 (dd, *J*=2.0, 1.1 Hz, 1H; Ar-H), 7.33 (t, *J*=1.8 Hz, 1H; Ar-H), 7.05 (d, *J*=1.8 Hz, 1H; Ar-H), 4.22 (dd, *J*=8.5, 6.4 Hz, 1H), 3.79–3.65 (m, 2H), 1.26 (s, 18H, *t*-Bu) ppm. ^{13}**C-NMR (100 MHz, CDCl$_3$):** δ_C= 196.48, 151.13, 136.61, 135.84, 135.65, 134.40, 131.45, 129.35, 129.29, 128.48, 127.38, 122.59, 121.83, 121.81, 121.76, 53.75, 35.59, 35.01, 31.55 ppm. **HRMS (APCI)**: *m*/*z* calcd. for $C_{27}H_{26}Br_2O$ $[M+H]^+$ 529.0561, found 529.0552.

4b: ^{1}**H-NMR (400 MHz, CDCl$_3$)**: δ_H = 8.72 (d, *J*=1.9 Hz, 1H; Ar-H), 8.25 (dd, *J*=1.9, 0.3 Hz, 1H; Ar-H), 8.06 (dd, *J*=1.7, 0.3 Hz, 1H; Ar-H), 7.88 (dd, *J*=1.7, 0.3 Hz, 1H; Ar-H), 7.74 (d, *J*=0.3 Hz, 1H; Ar-H), 7.51 (t, *J*=1.8Hz, 1H; Ar-H), 7.46 (d, *J*=1.8Hz, 2H; Ar-H), 1.39 (s, 18H, *t*-Bu) ppm. ^{13}**C-NMR (100 MHz, CDCl$_3$)**: δ_C = 183.03, 150.76, 141.46, 137.94, 135.12, 134.86, 134.56, 133.86, 133.72, 131.31, 131.26, 130.39, 124.37, 123.54, 123.08, 122.87, 121.92, 53.56, 35.12, 31.66 ppm. **HRMS (APCI)**: *m*/*z* calcd. for $C_{27}H_{26}Br_2O$ $[M+H]^+$ 527.0405, found 527.0416.

5,8-dibromo-2-(3,5-di-*tert*-butylphenyl)-2,3-dihydro-1*H*-phenalen-1-ol (**5**)

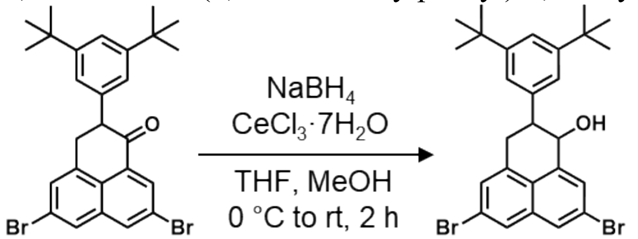

4a **5** 99%

Compound **4a** (0.100 g, 0.19 mmol, 1.0 eq.) and $CeCl_3 \cdot 7H_2O$ (0.120 g, 0.32 mmol, 1.7 eq.) were added to a dry flask Schlenk flask under Ar. Degassed/anhydrous methanol (5 mL) and dichloromethane (5 mL) were added. The solution was cooled with an ice bath and sodium borohydride (0.012 g, 0.32 mmol, 1.7 eq.) was added in one portion against Ar. The reaction

mixture was warmed to rt and stirred for 2 h. After quenching with an ammonium chloride solution and extraction with dichloromethane the organic phase was washed with water and brine and dried over Na_2SO_4. Evaporation of the solvent under reduced pressure gave product **5** as orange solid (100 mg, 99%). **^{1}H-NMR (400 MHz, $CDCl_3$)**: δ_H = 7.90 (d, *J*=1.9 Hz, 1H; Ar-H), 7.81 (s, broad, 1H; Ar-H), 7.61 (d, *J*=1.9 Hz, 1H; Ar-H), 7.49 (m, 1H; Ar-H), 7.39 (t, *J*=1.8 Hz, 1H; Ar-H), 7.18 (s, broad, 2H; Ar-H), 5.07 (t, *J*=3.4 Hz, 1H), 3.84 (dd, *J*=15.3, 12.4 Hz, 1H), 3.41 (dt, *J*=11.9, 3.5 Hz, 1H), 3.18 (dd, *J*=15.7 Hz, 4.0 Hz, 1H), 1.85 (d, *J*=4.0 Hz, 1H), 1.33 (s, 18H, *t*-Bu) ppm. **^{13}C-NMR (100 MHz, $CDCl_3$)**: δ_C = 151.45, 139.86, 138.86, 138.17, 135.97, 129.47, 129.32, 128.40, 127.18, 125.95, 122.43, 121.68, 121.25, 121.02, 72.36, 45.93, 35.08, 31.64, 29.80 ppm. **HRMS (APCI)**: *m*/*z* calcd. for $C_{27}H_{30}Br_2O$ $[M]^+$ 530.0639, found 530.0639.

5,8-dibromo-2-(3,5-di-*tert*-butylphenyl)-[2]triangulene **(1)**

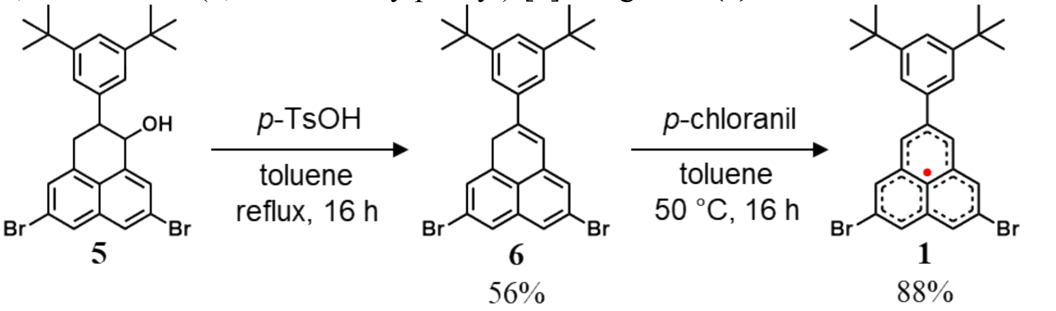

Alcohol **5** (200 mg, 0.38 mmol, 1.0 eq.) and *p*-toluenesulfonic acid monohydrate (*p*-TsOH, 21.5 mg, 0.11 mmol, 0.3 eq.) were added to a dry flask Schlenk tube under Ar. Anhydrous/degassed toluene (10 mL) was added and the flask sealed under Ar. The reaction mixture was stirred for 16 h at 130 °C while shielded from light. After cooling the reaction mixture, the solvent was completely evaporated under reduced pressure. The resulting solid was filtered through a short alumina plug (alumina, eluent: hexane/toluene (5:1)). The solvent was evaporated to give a blue-greyish powder (108 mg, 56%) that was used for the next synthetic step without further purification.

40 mg of the product mixture **6** (0.08 mmol, 1.0 eq.) and *p*-chloranil crystals (19.2 mg, 0.08 mmol, 1.0 eq.) were added to a Schlenk tube under Ar. Anhydrous/degassed toluene (6 mL) was added and the reaction mixture was thoroughly degassed *via* the freeze-pump-thaw method, and the flask was sealed under vacuum. The p-chloranil crystals dissolved slowly and the reaction solution turned dark purple with a greenish appearance when illuminated. The solution was stirred at 50 °C for 16 h. The cooled reaction mixture was quickly filtered through a short alumina column using *n*-hexane as eluent. Quick evaporation of the solvent gave **1** as blackish purple powder (35 mg, 88%) that was further purified by recrystalized from thoroughly degassed toluene to receive brownish needles. Alternative purification *via* sublimation ($p \sim 10^{-3}$ mbar, 230 °C) gave dark purple powder. **^{1}H-NMR (400 MHz, $CDCl_3$, 25°C)**: δ_H = 1.41 (s, 18H, *t*-Bu). **HRMS (APCI)**: *m*/*z* calcd. for $C_{27}H_{27}Br_2^{\cdot}$ $[M]^+$ 511.0455, found 511.0499; $[M+H]^+$ 512.0534, found 512.0531. Note: due to the open shell character of **1** only NMR signals related to the *tert*-butyl groups were observed at rt.

Nuclear Magnetic Resonance (NMR) spectra

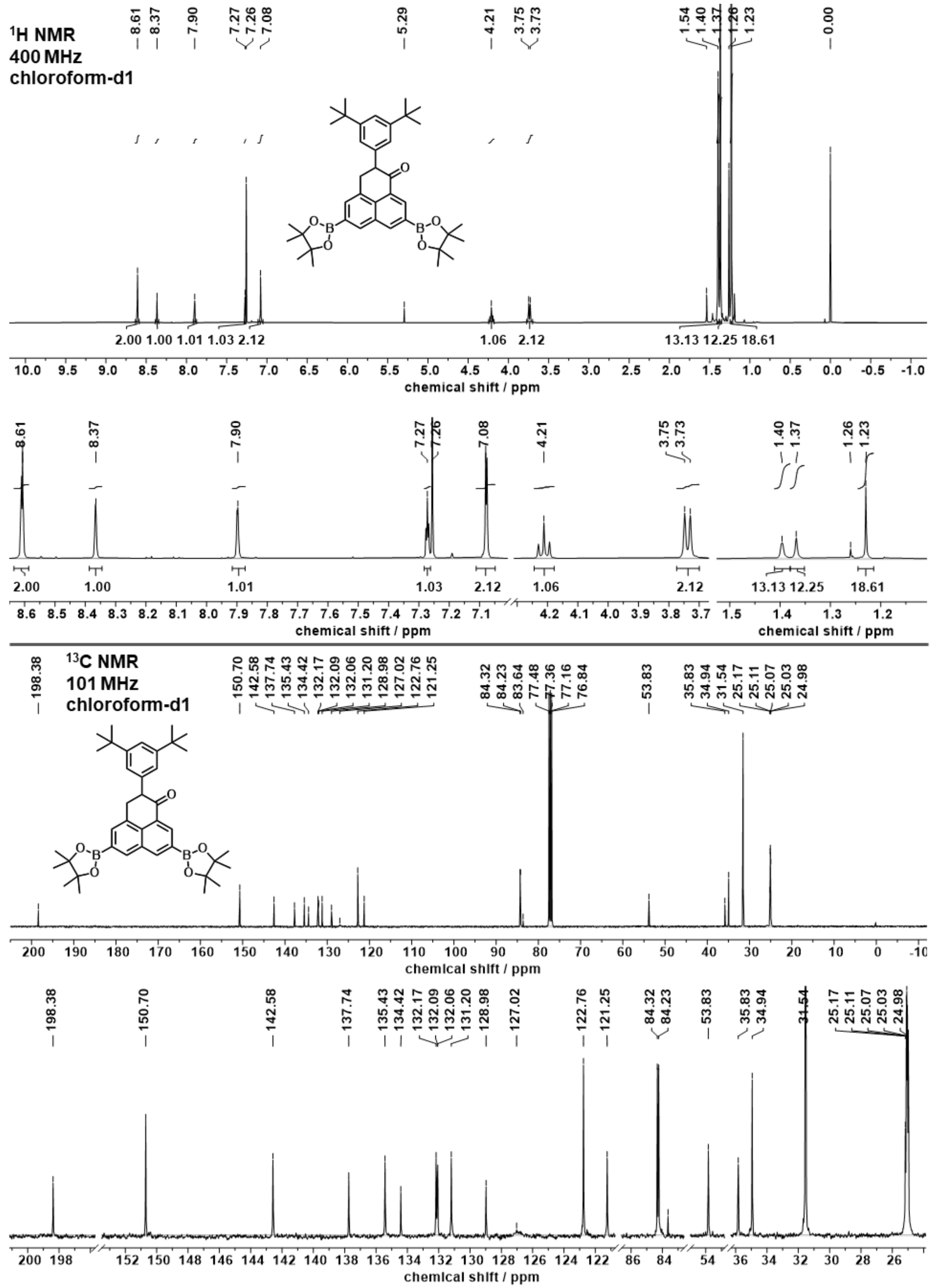

Figure S21. ^{1}H- and ^{13}C-NMR spectra of **3** measured in chloroform-d_1.

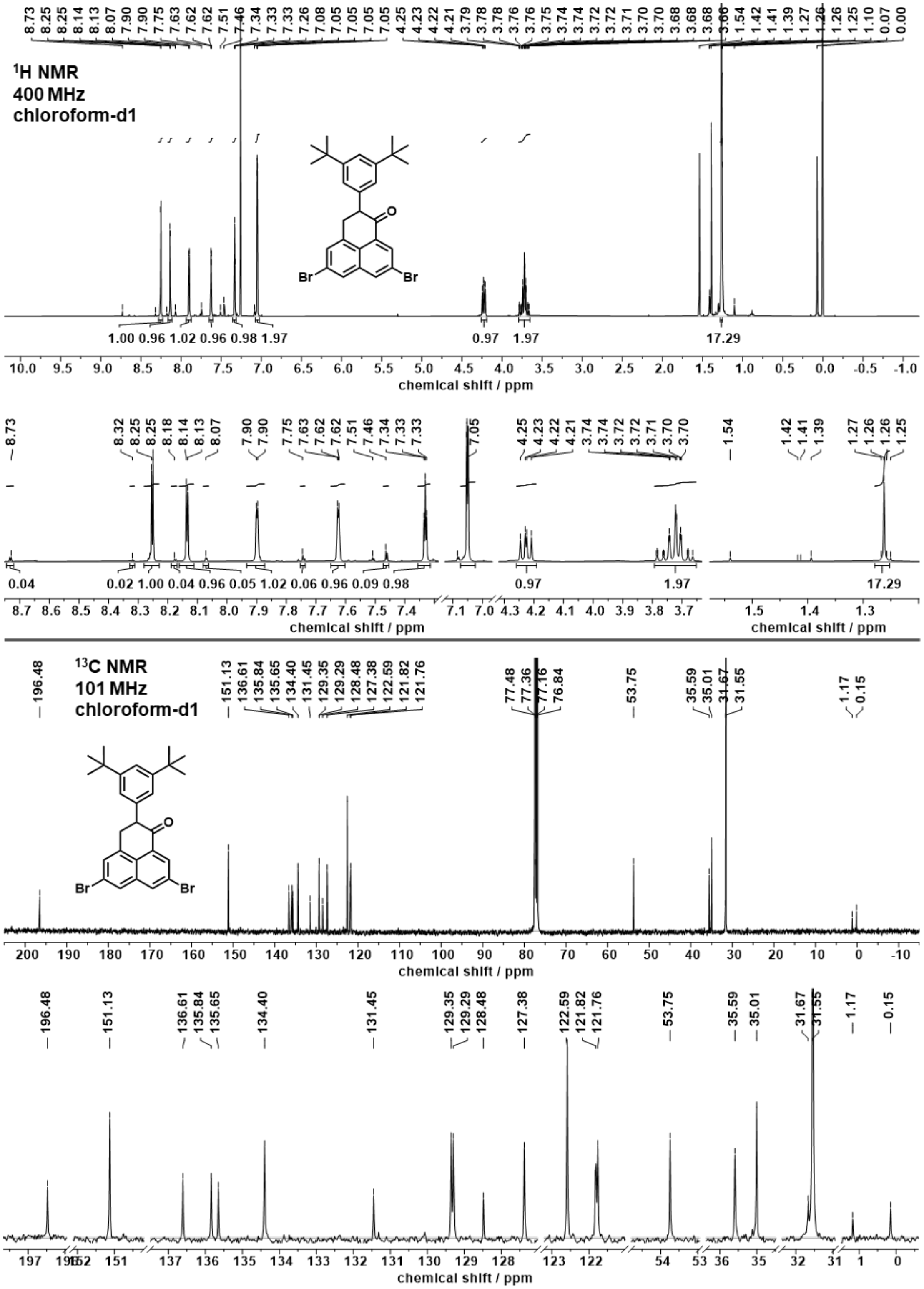

Figure S22. ¹H- and ¹³C-NMR spectra of **4a** measured in chloroform-d_1.

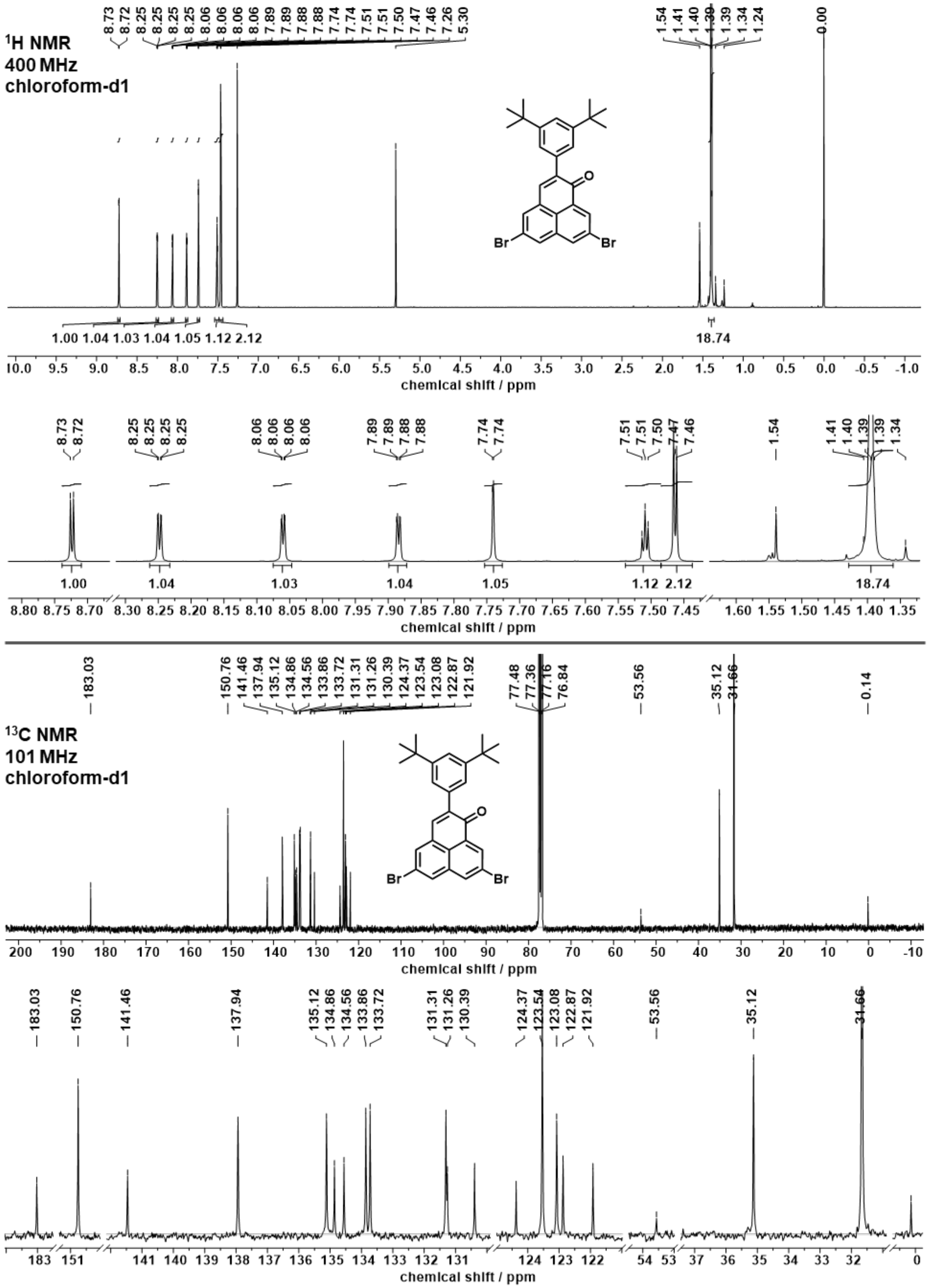

Figure S23. ^{1}H- and ^{13}C-NMR spectra of **4b** measured in chloroform-d_1.

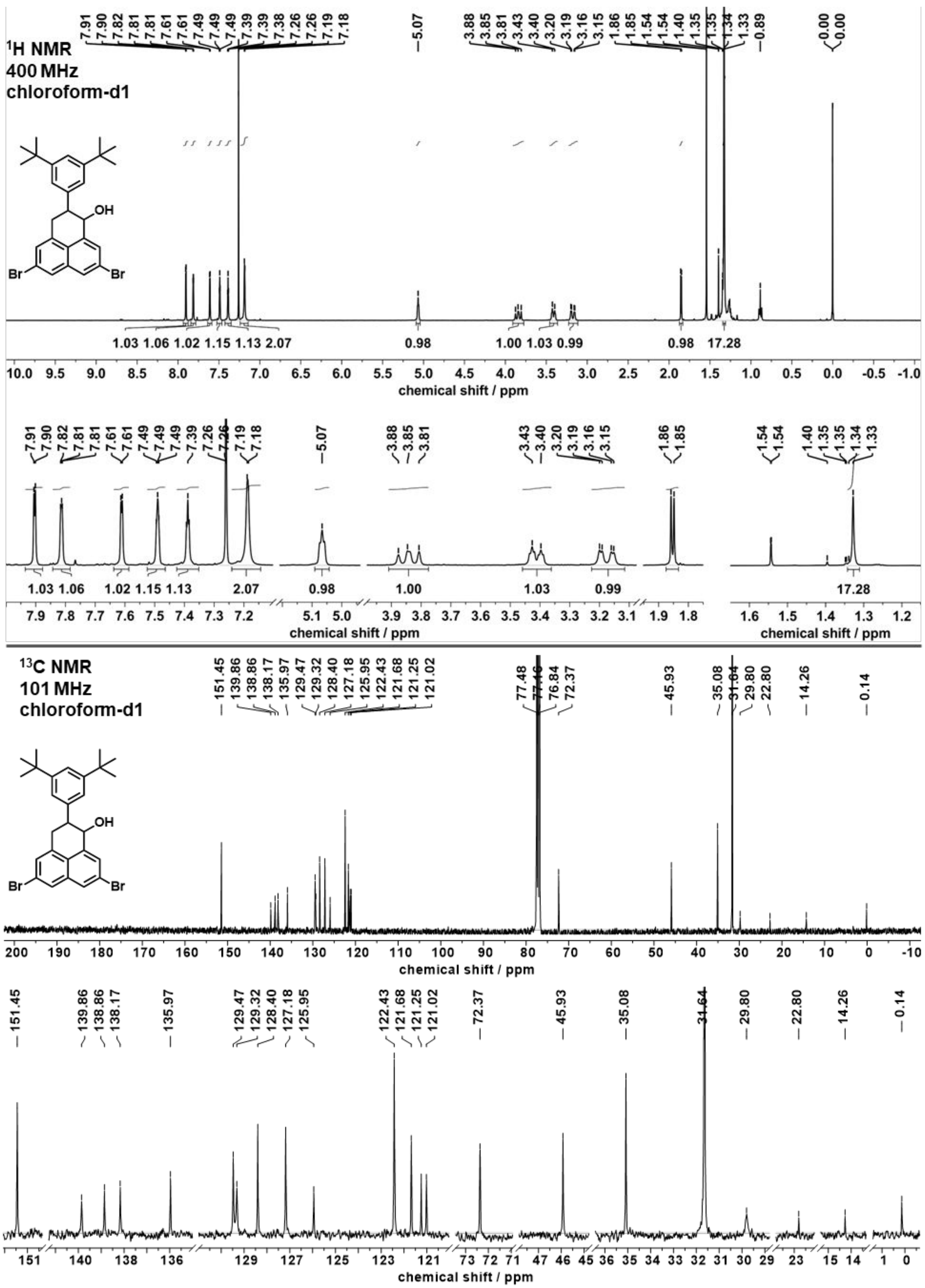

Figure S24. ${}^{1}H$- and ${}^{13}C$-NMR spectra of **5** measured in chloroform-d_1.

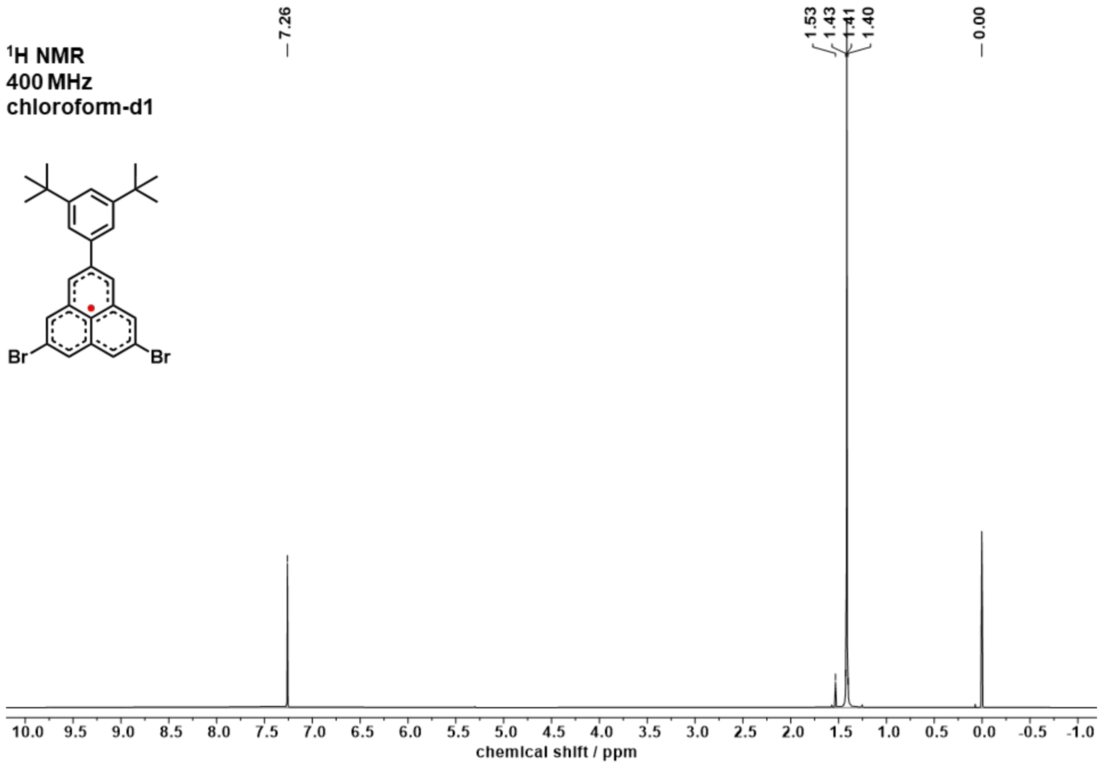

Figure S25. ^{1}H-NMR spectra of **1** measured in chloroform-d_1. Due to the presence of an unpaired electron, there is no signal derived from the ring protons of **1**.

Atmospheric-Pressure Chemical Ionization Mass Spectrometry (APCI-MS)

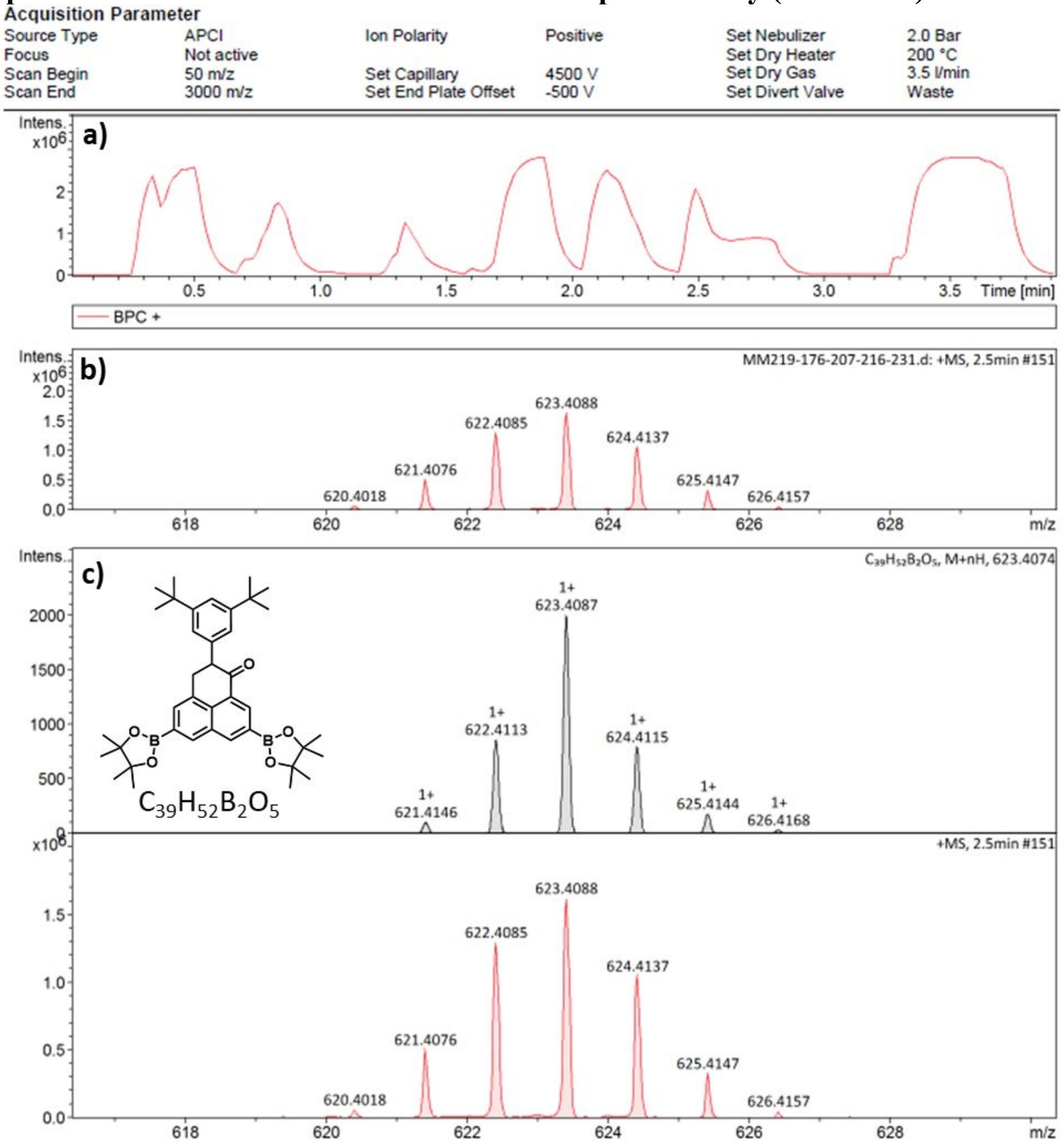

Figure S26. Results of high-resolution mass spectrometry (APCI-HRMS) for **3** showing (**a**) the base peak chromatogram, (**b**) the experimental peaks for **3** and (**c**) the comparison between simulated (gray) and experimental (red) isotope patterns.

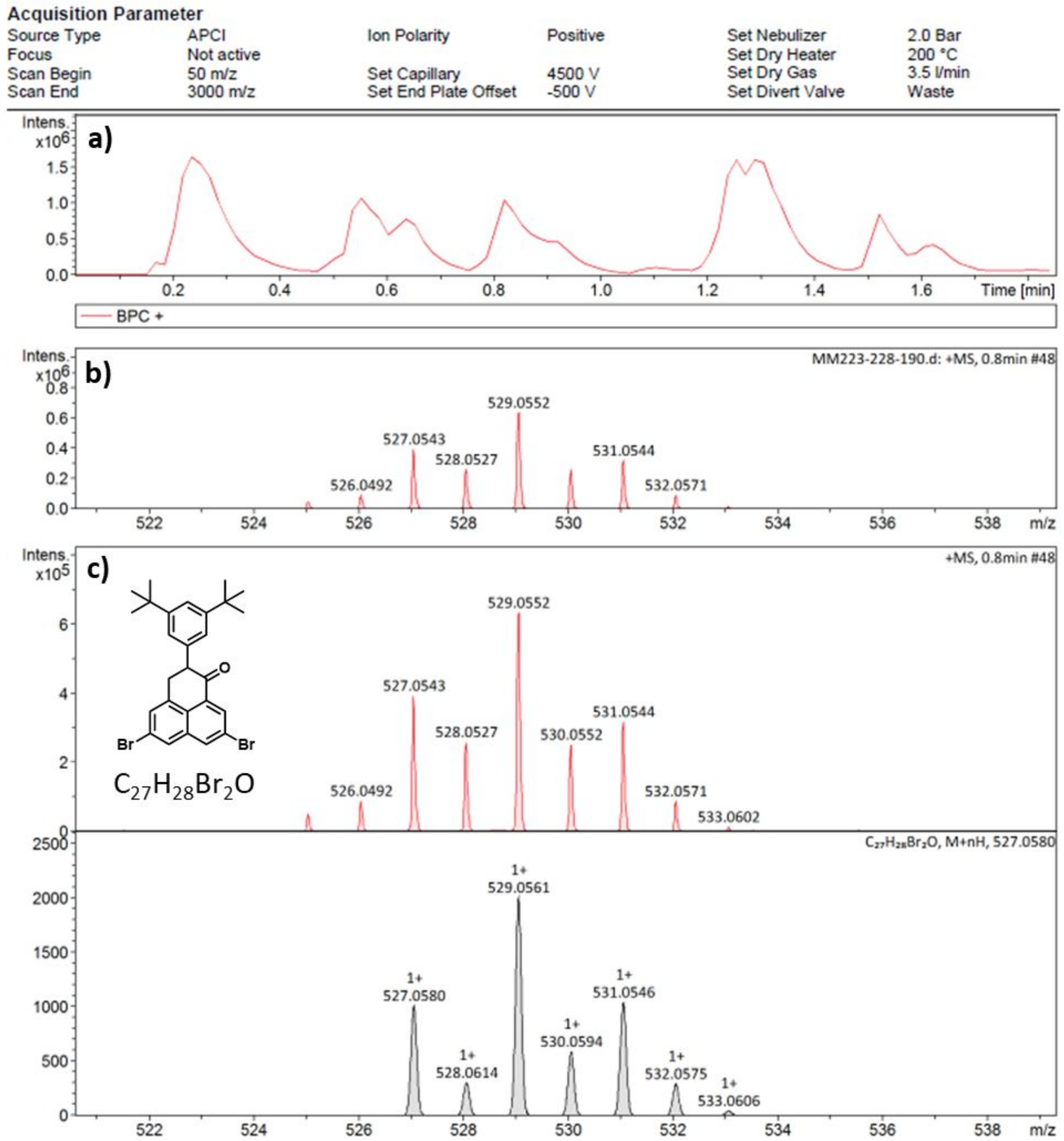

Figure S27. Results of high-resolution mass spectrometry (APCI-HRMS) for **4a** showing (**a**) the base peak chromatogram, (**b**) the experimental peaks for **4a** and (**c**) the comparison between simulated (gray) and experimental (red) isotope patterns.

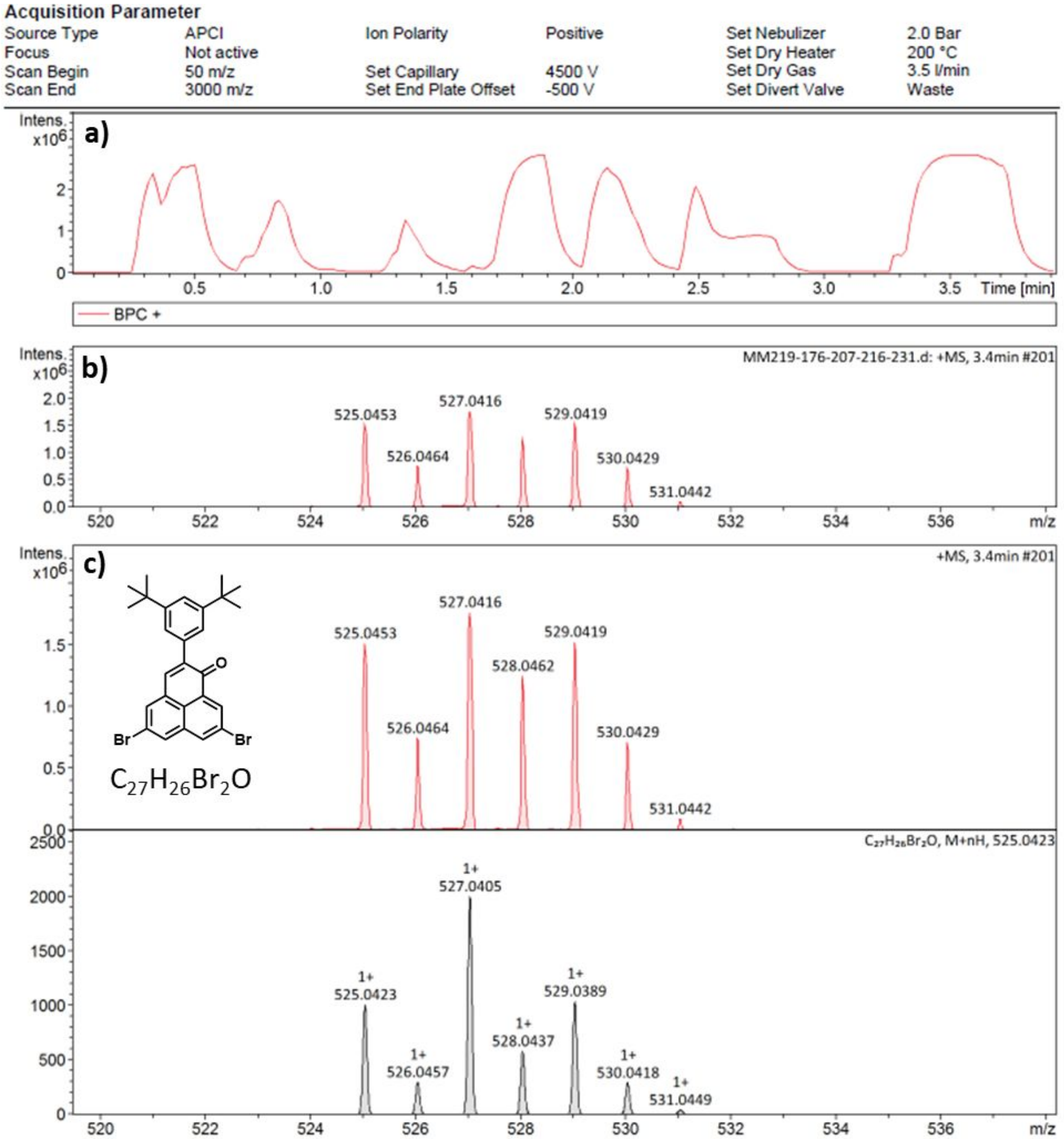

Figure S28. Results of high-resolution mass spectrometry (APCI-HRMS) for **4b** showing (**a**) the base peak chromatogram, (**b**) the experimental peaks for **4b** and (**c**) the comparison between simulated (gray) and experimental (red) isotope patterns.

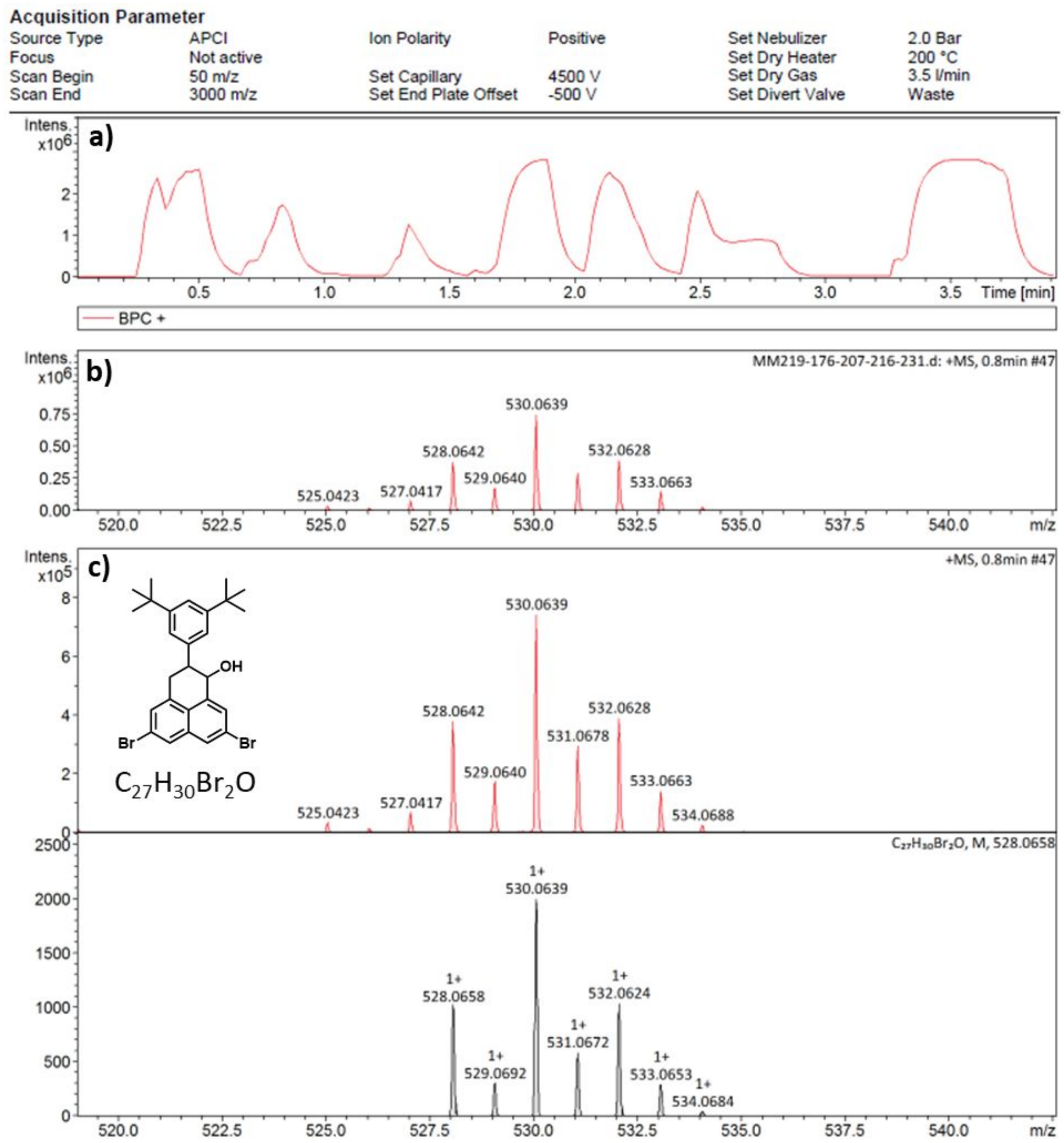

Figure S29. Results of high-resolution mass spectrometry (APCI-HRMS) for **5** showing (**a**) the base peak chromatogram, (**b**) the experimental peaks for **5** and (**c**) the comparison between simulated (gray) and experimental (red) isotope patterns.

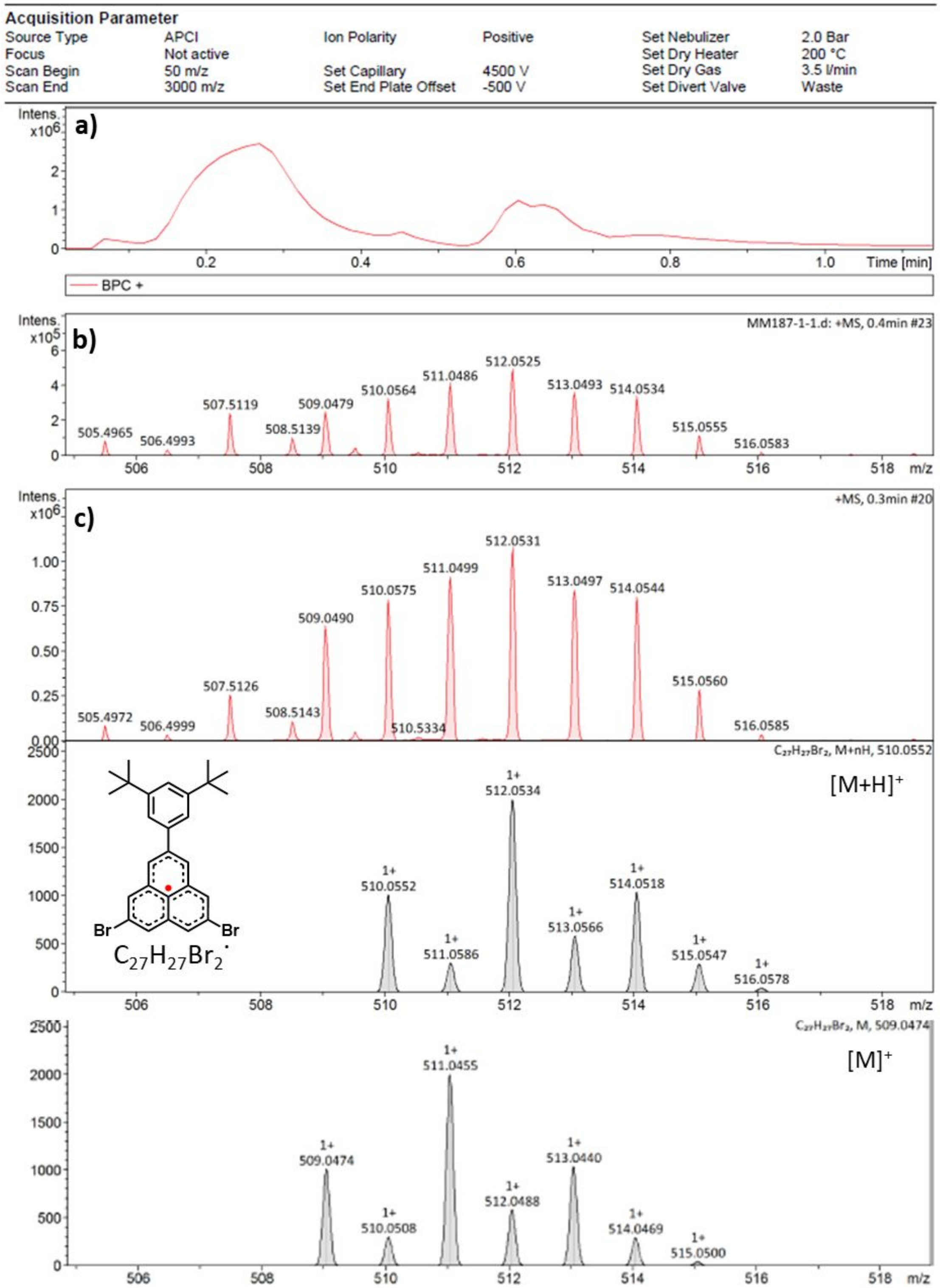

Figure S30. Results of high-resolution mass spectrometry (APCI-HRMS) for **1** showing (**a**) the base peak chromatogram, (**b**) the experimental peaks for **1** and (**c**) the comparison between simulated (gray) and experimental (red) isotope patterns.

Reference:

1. Uchida, K.; Mou, Z.; Kertesz, M.; Kubo, T. Fluxional σ-Bonds of the 2,5,8-Trimethylphenalenyl Dimer: Direct Observation of the Sixfold σ-Bond Shift via a π-Dimer. *J. Am. Chem. Soc.* **2016**, *138*, 4665-4672.
